# Extra-Limites

**Published:** 1838-07-01

**Authors:** 


### EXTRA-LIMITES.

### HOSPITAL REPORTS.

### I.

### Account of some remarkable Cases of Dropsy, with the Necroscopic Appearances. By Sir David J. H. Dickson, M.D. F.R.S.E. F.L.S. Physician of the Royal Hospital, Plymouth.

1st. *Thomas Smith,* æt. 31, R.M. of a sallow emaciated appearance, was a case of ascites; and very remarkable for the suddenness and rapidity of the effusion; and the speediness of its re-accumulation after tapping.

The swelling of the abdomen took place in one night, while he was on shore, on leave, from H. M. S. Royal Adelaide, and was, at first, supposed to arise from tympanitis (which is occasionally prelusive, as well as contemporaneous with dropsy), but it increased so rapidly, that on the fifth day it was necessary to have recourse to *paracentesis abdominis;* and between the period of his admission, on the 22nd January, and of his death, on the 19th July, 1837, this operation was repeated fourteen times. The following are the dates, and the pints of fluid (which, when tried, coagulated by heat) removed by each abstraction: viz. on the 27th January, 13lbs.; 8th February, 12lbs.; 16th February, 13lbs.; 5th March, 18lbs.; 19th March, 20lbs.; 7th April, 20lbs.; 23d April, 23lbs.; 9th May, 26lbs.; 19th May, 26lbs.; 27th May, 24lbs.; 7th June, 24lbs.; 16th June, 17lbs.; 30th June, 22lbs., and 11th July, 20lbs.; amounting altogether, to 278 pints; besides a very large quantity of fluid which escaped from the non-adhesion of the lips of the wound, after several of these operations. The renal secretion generally became pretty free afterwards; but decreased as the abdomen again enlarged; and it was almost suspended previously to the tappings; all of which he bore extremely well, except the last; after which the urine became very scanty, and dark, with much brick, or rush-coloured sediment; and he frequently vomited a brown, and ultimately a black coloured fluid before death, resembling melæna.

*Sectio cadaveris,* 31 hours P.M.—The body was withered, and sallow. The lungs were healthy; but adherent to the costal pleura, on the left side. The heart was twice its natural size, and the ventricles, especially the right, enlarged; but there was very little fluid in the pericardium. The abdomen contained several pints of serum of a deep yellow colour; though much had drained off through the last puncture. The great omentum, from the pressure of the fluid beneath, was, as it were, folded back, and adhered firmly to the diaphragm; and the peritoneum generally, was of a red, mottled colour; and its reflexions covering the small intestines, especially where they sink into the pelvis, and at the sigmoid flexure of the colon, were coated with a mixture of coagulable lymph, and pus. The liver was much indurated; and though it appeared to be shrunk *in situ,* was larger and heavier than natural, lobulated, and, internally, very pale, with a light yellow tinge, and its peritoneal tunic had a spotted, cribriform appearance, like the membrane closing the *foramen ovale,* and was of a semi-opake, yellowish-white, colour; dappled with purplish tints. The gall-bladder contained some thick, tar-like bile. The spleen was greatly enlarged, and much firmer than natural; and several of its arteries were ossified; which gave it, when cut, a gritty feeling. The stomach contained a fluid like soot and water; but its mucous coat, though soft, and that of the intestines were not, otherwise, changed. The kidneys were rather large and congested. A clot of blood, the size of a nut, covered with an evidently organized membrane, adhered to the side of the last puncture made by *paracentesis*.

2nd. *James Revitt*, seaman, æt. 44, was a still more remarkable case, from presenting a combination of, what might be called, antagonist disorders; for he was affected with dysentery, diabetes, and dropsy at the same time. He was admitted with severe dysentery, passing blood and matter, with mucous dejections. On the 18th February dropsy of the belly and limbs supervened; and, moreover, a diabetic flow of urine, amounting, at one period, to two or even three pots full of water, during the night. In this very extraordinary case the dropsical swelling of the limbs, scrotum, &c. assumed at one time, an intermitting character, if I may use the expression, by which I mean that, independently of position, the anasarca considerably increased and diminished; being much greater, not only on one day than another, but at different hours of the same day, which the patient attributed to the pumping, as he called it, or the violent straining by stool, and vomiting. But notwithstanding the quantity of fluid thrown off by these discharges, and which apparently exceeded the proportion swallowed, the serous infiltration of the limbs became so enormous, that on the 23rd July it was deemed necessary to make several punctures on the outside of the legs, below the knees; by the profuse discharge from which, he, for a time, experienced great relief. The dysenteric purging, however, continued unrestrained, by the most powerful astringents and opiates including nitrate of silver, sulphates of zinc and copper, &c. : the vomiting became more frequent; and, at last, the colour of soot; and to add to his misery, for a week before his death on the 10th August, the patient experienced the most distressing dysphagia; so that, at last, neither drink nor medicine could be swallowed; yet notwithstanding this complication of maladies, the extent of structural disease was found to be much less, *post mortem*, than had been anticipated. The occurrence of inordinate actions, through the influence of the nervous system, on different organs and structures, not only consecutively, but simultaneously, and in the latter cases producing, if I may so speak, antagonist functional excesses, according to the action, and reaction of various morbid sympathies, was here exemplified in a most extraordinary manner, and would afford a wide field for speculation "sed hypotheses non fingo." I shall proceed, simply, to detail the morbid appearances on dissection.

*Sectio cadaveris*, 14 hours P.M.—The cellular tissue, especially of the lower extremities, was loaded with serous infiltration; the thoracic organs were healthy. The abdominal cavity contained several quarts of fluid, but the peritoneum exhibited scarcely any change. The liver was normal in structure; and the spleen of the natural size; but some of the arteries had begun to be slightly ossified. The kidneys were much enlarged; and about twice their natural weight, while their internal structure looked paler than natural, and as if a fatty matter were deposited in their cortical substance. The whole alimentary canal, from the pharynx to the anus, was removed, and examined, but no organic lesion was discovered, to account for the severe fuliginous vomiting, and distressing dysphagia which had preceded death. The dysphagia seemed to have been spasmodic : for neither stricture of the upper part of the tube, nor any alteration of the mucous surface of the stomach, nor intestinal canal could be detected, until approximating the ileo-cæcal valve, where it became dark-coloured, and deeply and extensively ulcerated. These discolourations appeared in large patches of a leaden hue; and seemed to arise from the mucous and submucous tissues being impregnated with a carbonaceous matter, resembling *melanoma :* and they became darker, and the coats more diseased as they approached the extremity of the canal; where portions of its inner surface had assumed a blue-black colour, and felt rough, or gritty, and thickened, from the *melanotic* deposit. I have said

"resembling"—for whether this was an example of real melanosis, depending on the formation of a morbid product of secretion,—or, more probably, spurious, arising from the black discolouration of blood, from stagnation in the venous capillaries; or from chemical agency, I shall not take upon myself to decide; but a similar instance (amongst others) is adduced by Dr. Carswell, when, in severe chronic dysentery, the mucous and submucous coats were greatly thickened, and eroded; and the blood, poured into the cavity of the intestines, was converted into a black fluid, resembling liquid pitch; and the ulcers themselves were impregnated with blood similarly altered.

3*rd*. *John Tessimand*, R. M. æt. 22, was admitted from the Spanish battalion, on the 11th June, and presented a combination of ascites and hydro and pneumothorax. The symptoms were abdominal swelling, and tenderness arising from partial effusion and accretion. Great dyspnœa, and excessive action of the heart, which was forced quite over to the opposite side; so that its pulsations were distinctly seen and felt, not only under the right nipple, but all over the right side of the chest and back. He could sleep only on the left side; and, latterly, required to be almost erect. The lips became more livid, and the dyspnœa, at last, so urgent, that on the morning of the 11th August, he appeared to be in a state of asphyxia, and moribund, when *paracentesis thoracis* was performed at seven a.m., and thirteen pints of water were abstracted from the left cavity of the pleura. He experienced the greatest relief from the operation. The flow of urine increased, the heart gradually receded from its unnatural position, and he continued to improve until the 28th, after which the dyspnœa, and all the symptoms progressively increased; but no further operation could be attempted, and he died on the 5th of September.

*Sectio Cadaveris*, twenty-six hours p.m. On opening the thorax a quantity of gas escaped from the left cavity of the pleura; which was coated with yellow lymph; and the lung was bound to the mediastinum, by a layer of false membrane. The upper lobe was tuberculated, and much congested throughout. The right pleural sac contained several pints of fluid. The lung was healthy, but compressed into the upper part of the cavity by the serous effusion; and the pleura was thickened, and appeared, from a coating of lymph, as if covered with fine gauze. The pericardium contained four ounces of clear serum; but the heart, though rather large, was normal. The structure of the liver was soft; rather pale, and as if infiltrated with a watery fluid, which exuded on pressure; it adhered to the diaphragm; and the different viscera to each other; and to the abdominal parietes. The large intestines were tympanitic, and their serous tunic mottled, with numerous little spots, or granules of lymphy origin. The spleen and gall-bladder were normal. A slight effusion had taken place under the arachnoid membrane, which was rather opake, and a little thickened. The brain was firm, and healthy in structure; but the cerebellum was very soft; and a portion of serum flowed from the vertebral canal. I do not mean to enter into any detail upon the treatment of dropsy, which must be ever modified by the nature of the structure, and of the lesion of the organ obstructed, or congested; but, generally speaking, though not always, I have found it is necessary slightly to affect the mouth with mercury, before the due effect of other remedies, as diuretics, &c. could be obtained; and particularly when the liver is implicated:—but, while upon this subject, and without any reference to dropsy particularly, when it is oftener affected, I beg to observe, that, whatever may be said of its functional derangements, the liver is found to be much less frequently diseased, in our *post mortem* investigations, than from the writings of authors we should be led to expect. Indeed it may be stated, on the contrary, and although we are constantly receiving invalids from abroad, in all stages of thoracic and abdominal disease, that organic disease of the liver, comparatively speaking, is a rare occurrence.

With respect to the treatment of these cases: in that of *Smith*, great benefit was derived from hydrogogues, as croton oil, jalapine with colocynth, mercurial, and other purgatives; carminatives, calomel, or blue-pill and squills, with opium, or henbane, &c. digitalis, and other diabetic medicines, and drinks; such as the decoction of pyrola, with juniper, and bitartrate of potass. An infusion, in ale, of broom-tops, wild-carrot and mustard-seeds, weak gin punch, &c., a large draught of the latter, tepid, or of lemonade with a little spirit, and twenty-five, thirty, or more minims of the *liquor opii*—or, the muriate or other preparations of morphia, given in the forenoon, has been often followed by a free discharge of urine; when an opiate given at bed-time, excited *pervigilium;* and I may also remark, that when it has this exciting effect upon the sensorium, I have frequently given an anodyne draught in the morning instead of the evening, with advantage in cases of phthisis.

The pyrola I have often found useful in increasing the renal secretion, but sometimes inert, unless assisted by other means; which, indeed, is frequently the case with other diuretics. In the Second Case (Previtt) the treatment was rendered extremely difficult by the contra-indications, and the gastro-enteric irritability, and was, in a great measure limited to the vegetable and mineral astringents and sudorifics, as Dover's powders, and other forms of opium, in powerful doses, for iodine, mercury, &c. even by inunction, could not be tolerated.

The treatment of the Third Case was similar to that of the first except that purgatives were less freely, and anodyne and pectorals with the æthers, &c. more liberally administered. Elaterium, which is so powerful in reducing dropsical swellings, and gamboge, &c. were too sickening and drastic in these cases.

When I reported these instances to the physician-general of the navy, about six months ago, I stated that there were three other hydropic cases, then convalescent in this hospital; and in one of these at least, that I did not believe the relief could be of long duration; as it was complicated with asthma and other symptoms of organic disease. I however heard, lately, that he continued as well as when he was discharged. The others were dismissed cured; but with the caution that the disease would be liable to return from exposure to cold. One of them called upon me, sometime ago, for a prescription for a swelling of the face, from this cause; but it had not, then, extended to the extremities. The other resisted my wishes to be invalided; as I was convinced he was unfit to keep a night watch in Winter; and he, accordingly, was re-admitted on the 12th of January, affected with bronchitis, and general anasarca, but with so much irritability of the stomach and bowels, as to preclude the remedies most powerful in producing detumescence. Puncturing effectually removed the enormous swelling of the limbs, &c., but the relief was very transient. The stomach rejected everything; and he died on the 3d February.

Dissection revealed strong pleural adhesions, and much pulmonary, and bronchial congestion, and disease; but the abdominal viscera were nearly normal; with the exception of the kidneys, the cortical substance of which exhibited the degeneration of structure described by Dr. Bright; being indurated, and slightly granular, with great vascularity of the tubular portions, and papillæ, which appeared in red, isolated spots, on a brownish yellow ground. A very correct drawing of this morbid alteration has been taken by my first assistant, Mr. Weale, resembling the second figures in Plates 1st and 2nd of Dr. Bright's work. And I may, here, notice a still finer specimen of renal disease, in a patient, who had been a hard-drinker, admitted in the last stage of phthisis, and also with anasarca, though he made a large quantity of water. In this case the kidneys were found to be enormously enlarged: the right weighing thirteen, the left fifteen ounces. They were so much altered, as scarcely to retain any traces of their original structure; the cortical part being changed into a dull, white substance of semicartilaginous firmness; while the tubuli and papillæ were of a deep red colour, and the emulgent arteries were much dilated. I have already mentioned that I have generally found it necessary, at least slightly, to affect the mouth with mercury, before I could succeed in restoring the secretions and the action of the absorbents. I am aware that this opinion is not exactly in accordance with that of the last named, and other able physicians, who are averse to its exhibition in many cases, especially those with albuminous urine; but while I only state the result of my own experience, without entering further into the question, I readily admit that there are certain habits, and states of renal, and other forms of dropsy dependent on irremediable organic disease, in which, as in the case just alluded to, even the moderate administration of mercury would not only be useless but highly injurious. Upon the whole, the treatment of dropsy in this hospital has been very successful; but, I do not mean to say that such cases will be, frequently, permanent. Indeed I do not expect to meet with another case, similar to that of Lieut. Gahan, who has perfectly recovered, after having been twelve times tapped in 1833, as noticed in the numbers of the "Medico Chirurgical Review" for January and July 1834. I saw that officer, who brought a patient to the hospital, so lately as the 3d of December last. He had become rather corpulent; but continued in perfect health; although he had been arduously employed for several Winters, on the coast guard, and revenue service.

Royal Hospital, Plymouth,

7th March, 1838.

DAVID J. H. DICKSON.

---

### II.

### Surgical Report of the Dumfries and Galloway Royal Infirmary, for the Year 1836—37.

According to the practice of this hospital, for a long series of years, the Report embraces the period between 11th November, 1836, and 10th November, 1837. On the former date there remained on the books under treatment 22 surgical cases, and since then there have been admitted 207, making a total of 229. The daily average under treatment was $27\frac{3}{4}$.

In drawing up the following list, the average period of residence in the house, of each class of diseases, is struck from those cases only which terminated in recovery, and the average residence for all the cases cured was $44\frac{15}{68}$ days; this differs little from the average residence of every patient, whether cured or not, which was 46 days. The high average residence is to be attributed to the unavoidably protracted treatment in the cases of favus, necrosis, morbus coxarius, chronic impetigo, excema inveterata, cataract and burn. Of the whole number of cases there have been 161 cured; 6 relieved; 12 dead; 26 dismissed for other reasons; and 24 remain under treatment. From this it will be observed that the rate of mortality was 1 in $19\frac{1}{12}$, or rather more than 5 per cent. The whole amount of expenditure, divided by the number of patients admitted, gives £2. 4s. $4\frac{1}{2}$d. as the average sum which each patient costs the Establishment; and, if the sum be divided by the number of beds constantly occupied throughout the year, it will give £21. 15s. $6\frac{1}{2}$d. as the expence of each bed. But, if the expenditure be limited to the cost of provisions, medicines, wines, fuel, and miscellaneous charges, divided in the same manner, it will give for the first £1. 7s. $2\frac{1}{4}$d., and for the last £13. 7s., as the average sum which they have respectively cost. And, if the expenditure be further limited to the expense of salaries, servants' wages, and surgical instruments alone, it will be for each patient 10s. $5\frac{1}{4}$d. and for each bed £5. 2s. $11\frac{1}{2}$d.

| NAME OF DISEASE. | Male. | Female. | Average Age. | Average Number of Days in Hours. | Cured. | Relieved. | Remains. | Died. | Discharged for other reasons. |
|---|---|---|---|---|---|---|---|---|---|
| Abdomen, diseases of | | | | | | | | | |
| Fistula, Intestinal | | 1 | 16 | | | | | | 1 Incurable |
| Hernia, Femoral | | 1 | 73 | | | | | 1 | |
| Ditto, Inguinal | 1 | | 30 | 1 | 1 | | | | |
| Abscess, acute | 5 | 6 | 23 | 18 | 11 | | | | |
| Arm Drop | 1 | | 14 | 112 | 1 | | | | |
| Blood Vessels, diseases of. | | | | | | | | | |
| Absorbents, inflammation of | 2 | | 17 | 29 | 2 | | | | |
| Phlebitis, Chronic | | 2 | 31 | 8 | 2 | | | | |
| Popliteal Aneurysm (false) | 1 | | 30 | | | | | 1 | |
| Wound of Ulnar Artery | 1 | | 32 | 30 | 1 | | | | |
| Bones, diseases and injuries of. | | | | | | | | | |
| Caries | 2 | 1 | 70 | | | 1 | | 1 | 1 with advice. |
| Inflammation of | 1 | | 70 | 42 | 1 | | | | |
| Morbid Growth of | | 1 | 25 | 28 | 1 | | | | |
| Necrosis | 4 | 1 | 12 | 21 | 1 | | 2 | | 2 with advice. |
| Periostitis | 1 | 4 | 21 | 50 | 5 | | | | |
| Fractures. | | | | | | | | | |
| Compound of Cranium* | 1 | | 19 | | | | | | 1 by desire. |
| Ditto of Humerus | 1 | | 9 | 30 | 1 | | | | |
| Ditto of Condyles of ditto | | 1 | 18 | 41 | 1 | | | | |
| Ditto of Phalanges of Hands | 1 | | 37 | 55 | 1 | | | | |
| Simple of Fibula | 1 | | 21 | 29 | 1 | | | | |
| Ditto Fore-arm (both bones) | 1 | | 16 | 17 | 1 | | | | |
| Ditto ditto (radius) | 1 | | 34 | 18 | 1 | | | | |
| Ditto Malleolus Internus | 1 | | 22 | 26 | 1 | | | | |
| Ditto Patella (transverse) | 1 | | 40 | 45 | 1 | | | | |
| Ditto Ribs | 1 | | 66 | 18 | 1 | | | | |
| Ditto Sternum | 1 | | 55 | 30 | 1 | | | | |
| Ditto Thigh-bone, Neck and Trochanter of | | 1 | 74 | | | | | 1 | |
| Ditto Vertebra, 6th Cervical | 1 | | 32 | | | | | 1 | |
| With Wound Tibia | 1 | | 57 | 49 | 1 | | | | |
| Spine, Curvature of. | | | | | | | | | |
| Forwards | | 1 | 18 | 63 | 1 | | | | |
| Lateral | | 1 | 14 | 36 | 1 | | | | |
| Abscess, Lumbar | | 1 | 41 | | | | | | 1 incurable. |
| Eye, diseases of. | | | | | | | | | |
| Amaurosis | 1 | | 38 | | | 1 | | | |
| Cataract (double). | | 2 | 29 | 129 | 1 | | 1 | | |
| Conitis and Conjunctivitis | 1 | | 16 | 10 | 1 | | | | |
| Leucoma | 1 | | 21 | | | 1 | | | |
| External Injuries. | | | | | | | | | |
| Bruises | 3 | 1 | 44 | 19 | 4 | | | | |
| Burns and Scalds | 2 | 3 | 29 | | | | 1 | 2 | 2 irregular. |
| Frost Bite | 1 | | 19 | 28 | 1 | | | | |
| Wounds, incised | 1 | | 52 | 20 | | | | | |
| Lacerated | 3 | 1 | 41 | 73 | 4 | | | | |
| Ditto and Tetanus | 1 | | 27 | | | | | 1 | |
| Feigning Disease | 1 | | 19 | 44 | 1 | | | | |

* This patient had been trephined and was almost well, but we understand he died some time afterwards in consequence of fungus cerebri.

| | | | | | | | | | |
|---|---|---|---|---|---|---|---|---|---|
| Joints, Diseases and Injuries of. | | | | | | | | | |
| Anchylosis of Elbow | 1 | | 21 | | | | | | 1 Incurable. |
| Ditto of Shoulder | 1 | | 34 | | | | | | 1 ditto. |
| Cartilages, Inflammation of | | 1 | | 44 | 1 | | | | |
| Ditto, Ulceration of | 1 | 1 | 22 | 40 | 1 | | | | 1 by desire. |
| Hydrops Articuli | 6 | 1 | 26 | 19 | 7 | | | | |
| Ligaments, Inflammation of | | 1 | 43 | 24 | 1 | | | | |
| Morbus Coxarius | 3 | | 14 | | | | 1 | | 2 by desire. |
| Synovial Membrane, gelatinous degeneration of | 1 | 1 | 25 | | | | | | 2 by desire. |
| Dislocation of Humerus forwards | 1 | 1 | 32 | 2 | 2 | | | | |
| Sprains | 2 | 1 | 31 | 35 | 2 | | | | |
| Nervous System, diseases of | | | | | | | | | |
| Painful Tubercle on Spermatic Cord | 1 | | 39 | | | 1 | | | |
| Paraplegia* | 1 | | 30 | | | | | | 1 by desire. |
| Pelvis, Diseases and Injuries of. | | | | | | | | | |
| Fistula in Ano | 1 | | 22 | 18 | 1 | | | | |
| Ditto in Perineo, with Stricture | 2 | | 60 | 47 | 1 | | 1 | | |
| Spasmodic Stricture of Sphincter Ani | 1 | | 55 | 55 | 1 | | | | |
| Hemorrhoids | 1 | 1 | 21 | 17 | 2 | | | | |
| Hernia Humoralis | 2 | | 40 | 42 | 1 | | | 1 | |
| Hydrocele | 4 | | 57 | 35 | 4 | | | | |
| Mucous Membrane of Bladder, Chronic Inflammation of | 3 | | 23 | 43 | 2 | | | | 1 irregular. |
| Prostate, Enlargement of | 3 | | 65 | | | 2 | | | 1 by desire. |
| Sarcocele | 1 | | 29 | 124 | 1 | | | | |
| Urethra, Stricture of | 1 | | 27 | 145 | 1 | | | | |
| Scrofula | 3 | | 21 | | | | | | 3 with advice. |
| Sibbens | 3 | 7 | 22 | 28 | 8 | | | | 2 irregular. |
| Skin, Diseases of. | | | | | | | | | |
| Carbuncle | 1 | | 40 | 39 | 1 | | | | |
| Erysipelas, Thecal | 2 | | 42 | 28 | 1 | | | 1 | |
| Excema Inveterata | 1 | | 19 | | | 1 | | | |
| Favus | 3 | 3 | 13 | 165 | 3 | | 3 | | |
| Impetigo, Chronic | 1 | 1 | 14 | 69 | 1 | | 1 | | |
| Morbid Growth of Skin | 1 | | 17 | 111 | 1 | | | | |
| Onychia | 1 | | 30 | | | | 1 | | |
| Paronychia | 1 | | 25 | 21 | 1 | | | | |
| Psoriasis | 1 | | 48 | | | | 1 | | |
| Urticaria | 2 | 2 | 42 | 44 | 3 | | 1 | | |
| Syphilida | | | | | | | | | |
| Acne | 2 | | 27 | 44 | 2 | | | | |
| Bubo, Primary | 4 | | 30 | 45 | 3 | | | | 1 left. |
| Ditto, Secondary | 7 | | 27 | 34 | 5 | | 2 | | |
| Chancres | 4 | 1 | 21 | 29 | 5 | | | | |
| Condylomata | 2 | | 19 | 72 | 2 | | | | |
| Psydracious Pustule | 1 | | 32 | 56 | 1 | | | | |
| Rupea and Impetigo | 1 | | 27 | 147 | 1 | | | | |
| Ditto and Psydracious Pustule | 1 | | 22 | | | | | 1 | |
| Tubercles and Pains | 8 | | 30 | 36 | 8 | | | | |
| Ulcers | 5 | 3 | 28 | 43 | 7 | | 1 | | |
| Throat, Diseases and Injuries of. | | | | | | | | | |
| Ulceration of Pharynx | 2 | | 12 | 14 | 2 | | | | |
| Foreign Substance in Œsophagus | | 1 | 60 | 1 | 1 | | | | |
| Ulcers. | | | | | | | | | |
| Constitutional | 8 | 2 | 51 | 59 | 8 | | 1 | 1 | |
| Indolent | 9 | 4 | 33 | 40 | 11 | | 2 | | |
| Varicose | 4 | 3 | 43 | 78 | 3 | | 4 | | |
| Weak | 3 | 1 | 46 | 11 | 3 | | 1 | | |
| | 164 | 65 | | | 161 | 6 | 24 | 12 | 26 |

* This patient had extensive sloughings over the sacrum and nates, and his case was

The following cases are a few of those which have been considered the most interesting, and they are given in the order of the list. The remarks on them are made by Mr. Spalding and Mr. M'Lauchlan, the surgeons of this hospital.

### CASE OF FEMORAL HERNIA.

N—— H——, æt. 73, a pauper, admitted at 12 noon, 26th June, 1837, for a strangulated femoral hernia of the left side. Gives a very indistinct account of herself; but states that for several days past she had had a diarrhœa, for which she took a dose of the infusion of senna-leaf and Epsom salt on the 18th curt., and that on the night of the 23d, she observed the swelling in the groin; since then she has had no stool. Never had a hernia before.

*Symptoms.* 1st Local.—An oblong, smooth, and hard tumor, seated over Poupart's ligament, the neck of which can be distinctly traced to the saphenic aperture; it is tender to the touch, and the integument covering it is red. 2d General.—A constant burning pain at the epigastre; occasionally retching; hiccup; skin natural; countenance anxious; tongue loaded and brown, and moist; pulse 92, small and soft. After having unsuccessfully endeavoured to reduce the gut by the taxis, assisted by the warm-bath, warm water enemata, and, lastly, by an enema of tobacco infusion, the patient was placed on the operating table at 8, p. m., and Mr. M'Lauchlan divided the stricture at Gimbernat's ligament, so as freely to admit the finger into the cavity of the abdomen. The gut, however, could not be returned, owing to firm adhesions surrounding its neck. The gut itself had a dark brown appearance, and it was judged better to leave it, thus far relieved from the stricture, than to proceed to dissect off the adhesions. The edges of the wound were brought together and the patient placed in bed. A dose of castor oil was given, and an enema of salt and water again exhibited. The injection came away with a little feculent matter, but the oil did not operate. On the 27th, at 9, a. m., she had passed a bad night, and complained of pain at the stomach and retching, and of frequent ineffectual calls to stool; loaded tongue; pulse 64, intermitting and small; countenance anxious; extremities cold. Rept. enema. 12 Noon. The injection has come away with a little fæculent matter mixed with blood and mucus. ℞. Pil. colocynth. ct. iv. Ext. hyosc. gr. ij. M. ft. pil. iv. Pil. j. omni horâ cap. est. 6, p. m. No relief from the bowels; extremities cold and clammy; countenance more anxious; pulse 90. Vin. rubri. ℥ss. omni secundâ horâ cap. On the 28th, at 5, a. m. she vomited a quantity of green fluid and died.

*Autopsy,* at 5, p. m. The strangulated portion of gut was a link of the upper third of the ileum; its coats were mortified, and nearly perforated in one or two places. The mesentery attached to it was very much on the stretch, and it tightly bound down a great portion of the small intestine to the spine. The bowel above this part was loaded with fæculent matter and air, but below it the bowel was empty.

*Remarks.* It appears from the state of the parts, that incarcerated hernia had existed for some time, and it is likely the gut became strangulated from the effects of the diarrhœa, and the purgative medicine. The loaded state of the bowel above the incarcerated portion, and the condition of the mesentery, may

---

considered by the medical gentlemen who had attended him before his admission as one of diseased spine. On that account he was admitted into the surgical wards. When he left the house, however, ten days afterwards, there was reason to consider the case rather one of paraplegia than of diseased bone.

account for the continued obstruction. This obstruction might probably have been removed by a division of the adhesions, had the state of the mesentery been suspected; but this would have been attended with the risk of injury to the vessels, as well as to the gut which presented an almost gangrenous appearance.

The case of strangulated inguinal hernia was of the right side, in a male, æt. 30, and was reduced by the taxis.

### Case of Arm Drop.

A. D., æt. 14, baker, admitted 22d February, 1837, for loss of power of the muscles of the right shoulder-joint.

States that seven weeks ago he was seized with the influenza, and that since he has lost all power of the right arm; although he has otherwise regained his strength. He cannot assign any other cause for his complaint.

*Symptoms.*—While the arms are kept by the side, no perceptible difference is observed between the two shoulders, its sensation is perfect, and there is very little pain felt excepting on rather firm pressure applied over the brachial plexus. Of the different motions of the joint, he can only, in a very slight degree, perform adduction and rotation inwards, so that when the arm is raised, it falls powerless by the side. The motions of the other joints of that extremity are free and perfect.

He was ordered a solution of quina. A blister was applied over the brachial plexus, and the surface kept discharging by a repetition of the blister until the 9th of March, when there being little improvement from this treatment, he was directed to use the cold douche daily, and an embrocation of ol. tereb. and aq. ammo.—He continued under the latter treatment until the 4th of April, when the douche, embrocation, and solution of quina were omitted from there being little change of symptoms; and on that day he was again blistered over the brachial plexus; and the blistered surface dusted with gr. ¼ of strychnine, twice a day. On the 8th the strychnine was increased to a gr. ⅓ three times a day, and continued until twitchings were produced. On the 28th of May he was made an out-patient, very much improved, and subjected four times a week to a current of electricity through the affected limb, by which he, in a few weeks, perfectly recovered the power of it.

*Remarks.*—Various anomalous affections have been frequently observed to follow the influenza, apparently of a neuralgic character—was this a case of the kind?

### Case of Diffuse Popliteal Aneurysm.

J. M., æt. 30, a ploughman, admitted at 10 A.M., 29th August, 1837. Says that he was of a robust habit of body, that his habits had been dissipated, but that, until last May, he enjoyed good health, and never had any serious ailment, excepting once (three years ago) that he required general blooding and other antiphlogistic measures, for a cold he had caught after a debauch. States also that his mother died suddenly at the age of fifty, of a chest affection, of which she had complained for twelve months; and that his father died at an early age, but that he does not know of what complaint. He was their only child. About the beginning of last May, complained of rather severe pain, of a rheumatic character, in the left upper extremity, which stretched from the shoulder to the middle finger; it continued for nearly a week, and then quite subsided, and has never since returned. Eight or ten days afterwards, pain of a similar character commenced in the right lower extremity, and was felt especially severe in the groin and ham, and there were occasionally severe darting pains between these points. For rather more than a week he could only walk with the use of a staff, but the pain then abated so much that he was enabled to resume his avocations until six weeks ago, when the tumor was first observed in the poples, and since that time he has been bed-rid. The patient can assign no cause for the pain in the upper, and the commencement of the pain in the lower, extremity; but thinks the increase of pain in the latter, and the tumor, were produced by lifting a heavy weight two weeks previously to the appearance of the tumor.

*Symptoms.*—1st Local. There is a diffuse, fluctuating, pulsating and compressible swelling, occupying the right poples, and extending beneath the heads of the gastrocnemii, and the tendons on each side of the knee-joint; the pulsation is felt over every part of the swelling, and it ceases on compression of the femoral artery in the groin. The limb below the knee is slightly œdematous, and its sensation impaired; it sometimes feels hot, and at others cold, and is the seat of constant pain from the poples downwards, which is subject to violent and irregular paroxysms. The paroxysms, however, are most frequent during the night, and effectually prevent sleep—the patient declares that all the sleep he has had for the last five weeks, will not exceed six hours. 2nd. General. There is no cough, dyspnœa or palpitation, but frequent complaint of pain in the head. There are repeated and profuse perspirations; the body is very much emaciated; appetite impaired; bowels regular; tongue moist and clean; pulse 100, hard, full, and vibrating, but very compressible. The pulse of the affected limb has the same character as far as the tumor, but below it, there is no pulse to be felt; the pulse of the opposite limb is much less full and hard. Epistaxis has taken place three different times since the tumor was observed. Percussion duller over the cardiac region than natural, but especially so between the fourth and fifth ribs. Heart's impulse rather increased, and its apex strikes between the sixth and seventh cartilages; its two sounds can with difficulty be distinguished; the bruit de soufflet, is remarkably distinct over the whole of the anterior of the chest, and the bruit de rape equally so over the cardiac region. Along the spine also both are heard, but much less loud; and they are likewise very distinctly heard in the tumor itself.

States that he has at three different times been freely blooded generally, and had leeches and seven blisters applied to the tumor.

℞. Muriat. morph. gr. i., aquæ ℥i., acid pruss. gtt. j., M. ft. haustus, statim sumendus. 4 P.M. A short sleep from the anodyne; bowels have moved; no other change. After consultation, Mr. M'Lauchlan tied the superficial femoral at the crossing of the sartorius. The pulsation in the tumor immediately ceased; the pain of the limb was greatly lessened, and the pulse became softer. Rept. haustus. 6 P.M. No sleep; no pulsation in the aneurysm; leg and foot of a natural heat, but to the patient they feel "burning;" no pain; pulse 100, and continues softer. 11 P.M. Toes becoming rather cold; no other change. The toes to be rubbed with the hand.

30th, 9 A.M.—Has had some sleep and altogether a better night's rest than he has had for the last six weeks. Since 4 A.M. has complained alternately of heat and cold in the limb from the knee downwards, but chiefly of heat; the toes still feel to the hand cold, and the cold is spreading up the foot; sensation not improved in any part, and the toes and dorsal aspect of the foot are barely sensible to pinching. There is some swelling around the wound, its edges adhere at one or two points; from its upper end some glairy fluid escapes. Pulse 102, rather harder; above the ligature the artery throbs violently; below it, there is no pulsation; countenance anxious; less of the bruit de soufflet; other stethoscopic indications as before. Continue the rubbing. 11 A.M.—Upper part of the foot not so cold; no stool; tongue moist. Pil. Rhei ct. iii. 6 P.M.—No stool; no pulsation below the ligature; toes still cold. On a deep inspiration there is pain at the upper part of the sternum; pulse 106. Enema com. statim. 11 P.M.—Bowels freely moved. Rept. haust. anodyn.

31st.—Some sleep; complains less of the sensation of heat and cold in the leg; pain continues at the sternum, and at 4 A.M. he felt, he says, a sensation there as if "a horse were galloping." Toes still cold, and the upper part of the foot again became so; from the wound there is some healthy pus discharged, and around its edges a slight degree of redness; no pulsation; no stool; tongue moist; pulse 100, and softer; has not perspired since the operation. Simple dressing to the wound. 1 P.M.—No change. ℞ Tart. antim. gr. i, aquæ ℥vi. solve; ℥ss. omni secundâ horâ cap. est. 6 P.M.—Bowels have moved; pulse 102, cont. medicament. omni horâ. 11 P.M.—The "burning" sensation in the leg and foot returned; toes and foot still require to be rubbed. Rept. haust. anodyn.

September 1.—A good night's rest; has feeling of "burning," and no pain of the chest; less anxiety of countenance; pulse 100, and more soft; perspired profusely; tongue moist; no stool. More redness around the wound, and an increase of the discharge. No other change. Continue. 5 P.M.—No stool; pulse 114. Pil. rhei ct. iii. et rept. haust. anodyn. h. s.

2d., 9 A.M.—Had some sleep, but started during it; and he does not feel so well to-day; no stool; tongue dry; pulse 114, and still soft; perspired less. From the wound there is rather a copious discharge; coldness extends up the foot. Enem. com. statim. 6 P.M.—Bowels have moved freely; tongue moist; pulse 116. The articulars of the inner side of the knee-joint can be seen and felt to pulsate; took dinner, and he feels happier since this was observed. Rept. haust. anodyn.

3d., 9 A.M.—A better night, and he has taken breakfast pretty well; pulse 116; no stool; tongue moist. The internal articulars are still felt pulsating, but less strongly; there is no pulsation in the aneurysm, which is not diminished in size since the femoral was ligatured, and it feels rather hot and is acutely painful to the touch. The coldness continues in the foot, and the skin covering it has a mottled appearance; the discharge from the wound is still rather plentiful. Ol. ricini ʒvj. statim. 1 P.M.—Hemorrhage took place from the upper end of the wound; the blood appeared to be venous, and he may have lost between two and three ounces. Slight compression applied over the artery. 6 P.M.—No recurrence of the hemorrhage; one free evacuation from the bowels; pulse 110. 11 P.M.—Sleeping.

4th., 8 A.M.—Slept nearly the entire night, but the sleep was disturbed by starts and dreams; he does not feel so easy to-day, and attributes this in part to the pain of the wound; no hemorrhage, no stool; tongue moist; pulse 110; coolness extending up the leg. 1 P.M.—The compress removed from the wound; its edges seen gaping, and from it some ill-conditioned bloody pus escaped. Simple dressing. ℞ Tart. potass. et sodæ ℥iss. aquæ ℥viii. solve; ℥iii. statim, et ℥ss omni horâ donec alvus respondeat. 6 P.M.—No stool; occasionally hiccup; pulse 114. ℞ Muriat. morphiæ gr. j. hyd. c̄ creta gr. iv. M.

5th., 8 A.M.—An indifferent night, but he feels rather easier to-day, and inclined for breakfast; has taken all the saline solution without relief from the bowels; pulse 110; no hiccup; perspired a little; a flush on the right cheek. The symptoms of mortification increase; the poples is more swelled and hot, and a slough is observed to be forming on its outer side; wound looks better and the pus discharged is more healthy. Dressings continued. Bladders filled with hot-water applied around the leg. Enema com. statim. 6 P.M.—Very uneasy and complaining of intolerable pain or heat in the thigh, and belly as far up as the sternum, which is increased by pressure; the belly feels hot; bowels relieved; stools natural; tongue moist; hiccup; pulse 130. Rept. haust. anodyn. statim. Foment. calid. abdom.

6th, 8 A.M.—The anodyne draught was repeated at 4 A.M., he has slept a great part of the night and is still disposed to sleep ; pain of abdomen relieved ; tongue moist, pulse 115 ; hiccup ; mortification spreading. 11 A.M.—Amputation proposed, but the patient would not consent to it, " at least to-day." 6 P.M.—Pulse 100 ; no stool ; tongue moist ; no change. Rept. sol. salina.

7th., 5 A.M.—No change. Patient consents to have the limb removed, but just when he was about to be lifted from the bed he died from terror.

*Autopsy thirty hours after death.*—The ligature had separated, and both ends of the artery were filled by a coagulum ; the aneurysm was diffuse, and its sac formed by the surrounding tissues. The diseased portion of the artery was exactly opposite the bend of the joint, and the aperture in it sufficient to admit a large pea. The coats of the artery when tied were healthy. The heart was removed, with a very small portion of the aorta, and the following is the state of that organ—its right side normal ; great dilatation of the left ventricle and hypertrophy by extent of surface ; on the mitral valve next the aorta, is an aneurysm about the size of a walnut, which projects into the corresponding auricle, and is perforated by three openings, all of which are sufficiently large to admit a garden pea ; two of them open into the auricle, and the other perforates the mitral valve, and opens into the ventricle. The aortic valves are completely defaced by deposites of calcareous matter, which project and hang into the ventricle. Osseous deposites beneath the lining membrane of that portion of the aorta removed.

*Remarks.*—This case has many points of interest to the practical surgeon, and is one, among many, which proves the necessity of minute enquiry into the state of the internal viscera in such cases. Without the aid of the stethoscope the diseased state of the heart could not even have been suspected ; none of the general symptoms, with the exception of the headache and the pain in the left arm, which latter occurred sometime before the aneurysm indicated any disease of that organ. The patient also positively declared that he never had any symptoms of an affection of the heart, and the character of the pulse too seemed to contra-indicate any valvular disease. With regard to the best mode of treatment in such cases, there can be little doubt that amputation is the only remedy likely to prove successful—the diffuse nature of the aneurysm and the want of pulsation below it, naturally suggested such a proceeding, had not the man's * repugnance to the removal of the limb precluded it. Subsequently, when this operation was considered indispensible, from the symptoms of mortification supervening, it may be matter of surprise that it was not proposed at an earlier period, but the return of the circulation in the articulars, and the continued aversion of the patient to the removal of his limb, were the causes of delay ; yet even had the operation been proposed to him sooner, it is probable from the actual result, that the mere idea of such a measure would have been too much for his timid nature. It will be observed by the report that symptoms of disease of the heart supervened on the day subsequent to the operation, and continued daily to increase—these symptoms it may be naturally inferred became prominent in consequence of the greater resistance to the circulation caused by the ligature on the artery.

The autopsy was conducted by that able anatomist, Dr. Alexander Jardine Lizars, of Edinburgh, who happened to be in this neighbourhood, and to whom we have much pleasure in expressing thanks for his valuable assistance ; and it may be right to add that that gentleman was also of opinion that amputation was the only remedy. Further examination of the arterial system was prevented by

---

* Before he was admitted into the Infirmary, the removal of the limb was proposed to him, but he positively refused to submit to it, and only consented to become a patient there with the understanding, that the vessel only would be secured.

the objections of the relatives of the man, who had, to prevent any examination, removed the body home seven miles from town. on the day of his death, so that the examination had to be conducted there the following day in presence of several relatives, after a partial consent had been obtained.

### Case of Wounded Ulnar Artery.

D. N., æt. 32, labourer, admitted 9th of August, for an incised wound across the left wrist. The wound penetrates to the bone, dividing the tendons and ulnar artery; it was produced accidentally, eight days ago, while cutting a stick, and has on three different occasions bled profusely. Graduated compresses and a bandage applied. Saline solution. The wound suppurated in a few days, and gradually healed with impaired motion and sensation of the ring and little fingers.

### Case of Fractured Phalanges, &c. Amputation and Cure.

J. D., a male, æt. 37, labourer, admitted the 17th of June, 1837, at 6 P.M.—He received the following injuries from the bursting of a stone, he was engaged blasting at a quarry, about an hour-and-half before admission; and working at the time on the edge of the quarry, he sustained a fall of twenty feet.

All the fingers of the right hand were dreadfully lacerated on their dorsal aspect; their joints exposed, with comminuted fracture of the first or proximal phalanges, and fracture of the phalangeal extremity of the metacarpal bone of the right little finger. The bones of the thumb were sound and its apex slightly lacerated. The ring and little fingers of the left hand, and the portion of the palm of that hand corresponding to these fingers, were lacerated and fractured in a similar manner; and there were wounds of less importance on the other fingers, as well as on the fore-arm of that extremity. The face and neck were blackened with powder, and studded with punctured wounds. The coats of the right eye were torn and sunk, in consequence of the humours having escaped. The vision also of the left eye was nearly lost from injury done to the iris and its other coats. From the wounds of the face he suffered pain, and felt cold. Pulse was 60. He stated that he was of a very healthy constitution and that wounds rapidly healed with him.

Sand and earth were removed from the different wounds, the eyes syringed and cleansed, and warm fomentations applied. Heat was applied to the feet and warm drink given him.

At 8 P.M., Mr. Spalding and Mr. M'Lauchlan proposed amputation of the injured fingers, but the patient would not submit. On the 18th at 8 A.M., he had passed a bad night; face swelled; no increase of pain; skin hot; tongue foul; no stool; pulse 70 and sharp. ℞. Mag. sulph. ℥j. tart. antim. gr. ¼. aqua ℥viij. solve; capiat statim. At 1 P.M., after a consultation with the medical officers of the institution, amputation was again proposed, to which the patient now agreed. Mr. M'Lauchlan removed the fingers of the right hand by dividing the phalangeal extremities of all the metacarpal bones—the flap being taken from the palmar surface. The ring and little fingers of the left hand, with portions of the corresponding metacarpal bones, were also removed; the flaps of both brought together by the interrupted suture, and cold cloths applied. Very little blood was lost, and the patient bore the operations well. The wounds suppurated, but by the 25th of July they were both healed, as were also the different puncture wounds of the face and neck. On that day the right eye was completely sunk in its orbit, and with the left eye he could just perceive the light; lymph appeared to be effused between the layers of the cornea, and the anterior chamber was occupied by an opaque substance; the pain of the eye was more acute, and one or two large vessels were observed ramifying towards the cornea. Leeches were several times applied to the angle of this eye and the extract of belladona rubbed twice a day on the eyelids. He took two grains of calomel and a quarter of a grain of opium night and morning, until the 7th of August, when his mouth became sore. The inflammatory symptoms had then subsided; the opaque substance was in a great measure absorbed; the cornea was clearer but with the lower edge of the iris adhering to it, the pupil was of course irregular, and the vision, although improved, was still imperfect. For two or three days previously to the last date he had complained of a smarting in the cicatrices, and on that day matter was discharged from the cicatrix of the right hand, and part of the adhesions had been absorbed. The calomel and opium were omitted, and the sulphate of zinc solution applied to the wounds; they speedily healed, and on the 10th August he was dismissed at his own desire, in the above condition.

*Remarks.*—With the thumb and the remaining portion of the right hand, the patient could use a spoon and other articles, and assist himself in many other ways: the left hand was of course of still more use to him.

### Case of Transverse Fracture of the Right Patella.

J. V., æt. 40, a male, admitted 17th September, 1837. The injury was occasioned by a fall on the knee. A roller was applied from the toes to the patella and the limb extended on a straight splint, with the foot elevated. Pieces of bandage were placed on both sides of the patella, and confined there by several turns of a roller applied both above the condyles of the femur and below the head of the tibia; the ends of the lateral bandages were then drawn together and tied so as to bring the fractured extremities into as close apposition as possible. It was found, however, totally impossible to make the patient, who was of a very irritable temperament, submit to the necessary restraint, or to keep the two portions of bone in apposition, notwithstanding of unremitted efforts for that purpose, and to make him comprehend the necessity of repose. He was consequently dismissed on the 14th November, with the fractured extremities separated for rather more than an inch, and united by ligament; the motion and strength of the joint being impaired.

### Case of Fracture of the Neck and Trochanter of the Right Os Femoris.

J. K., a female, æt. 74, was admitted 1st June, 1837, for an injury, the result of a fall on the street, the preceding evening. She died four weeks after admission from diarrhœa, and extensive sloughing over the sacrum.

*Autopsy.*—The neck of the os femoris was broken from the shaft of the bone, and forced into the substance of the great trochanter, which it had split. A bridge of new bone had formed between the posterior portion of the great and little trochanters; there was no osseous union of the neck, and but slight adhesions to the surrounding parts by a ligamentous substance.

*Remarks.*—This is another example of the improbability of osseous union taking place in fractures of the neck of the femur.

### Case of Fractured Cervical Vertebra.

T. C., æt. 32, admitted 3d December 1836, at 6 P.M. He had been sitting on the box of a cart five days ago, and the horse suddenly and unexpectedly stopping, he fell from the cart upon the back of his neck and shoulders. He was carried home senseless and paralyzed, and continued so for four and twenty hours; after which he became sensible, but the paralysis remained: the stools had been voided without control, and the urine required to be drawn off. He had taken several doses of purgative medicine and was blooded generally the day preceding his admission.

When admitted he complained of pain from the back of the head to the shoulders, principally however at the upper part of the neck, much aggravated by motion, and pressure gave the most acute pain over the situation of the 2nd, 3rd, 4th and 5th cervical vertebra. The lower extremities were completely paralyzed, the upper ones nearly so, and both were void of sensation. The integument covering the abdomen could likewise be pinched without giving the least sensation. The integument of the thorax was slightly sensible, and of the neck and face perfectly so. The respiration appeared to be performed entirely by the diaphragm; the air-tubes were obstructed by mucus, and there appeared to be no power to discharge. Pulse was 64 and compressible. Thirst; skin cool; repeated short rigors; two stools voided involuntarily since his admission; hypogastrium distended. Urine drawn off, and leeches to be appplied to the neck. He died suddenly at 9, P.M.

*Autopsy* 12 *hours after death.*—There was a fracture of the right side of the arch of the 5th cervical vertebra running obliquely downwards and outwards, into the articular surfaces of the 5th and 6th vertebra. There were slight adhesions between the left pleura, and the air-tubes contained much mucus.

*Remarks.*—Death doubtless resulted, in this case, from compression of the spinal marrow. Had the nature of the accident been ascertained, and all motion of the neck prevented, it is possible the patient might have recovered. Here the anatomy of the parts accounts distinctly for the symptoms; there was sensation and motion in those parts only supplied by the cerebral nerves and cervical plexus, which come off above the seat of injury; and the superabundant mucus in the air-tubes seemed to be the result of the diaphragm being unable, unassisted, to discharge that natural secretion.

### Case of Lateral Curvature of the Spine.

S. P., æt. 14, a servant girl, admitted 28th August, 1837. During the early part of the Summer she had had an attack of influenza, and nine weeks before admission she felt pain in the back, and five weeks afterwards the curvature was noticed. On two different occasions six leeches had been applied to the pained part of the back, and afterwards the tartrate of antimony ointment: from the latter application some relief was experienced. She was permitted to be out of bed during the day.

At her admission the curvature was very considerable, and it involved the nine lowest vertebræ; there was very little pain in the recumbent position; but the erect posture, more particularly in walking, produced pain, and pressure made over these vertebræ had the same effect. General loss of flesh; natural sleep; bowels regular; tongue moist; pulse 96. She was directed constantly to maintain the recumbent position; was on four different occasions cupped to a moderate extent over the seat of pain; allowed a nourishing diet, and the bowels were regulated by aperient medicines. On the 4th October she was dismissed with the spine nearly straight.

*Remarks.*—The curvature in this case apparently arose from the debility occasioned by the influenza; and the improvement may be attributed to strict attention to the recumbent posture, and the generous diet. We have no doubt the curvature would have been completely removed, but the patient would not submit longer to the treatment.

### Case of Cataract.

J. C., a female, æt. 29, admitted 30th November, 1836, for double cataract. The cataract of the left eye had commenced twelve years, and that of the right eleven years, before her application. For ten years she had been totally blind with the former, and nearly so with the latter eye. Both cataracts commenced with a mistiness before the eyes, with headache, vertigo, and dyspeptic symptoms. Both, but more particularly the right one, had a dirty brown appearance. She could not assign a cause for the disease, and it was not hereditary. There appeared to be nothing to contra-indicate the operation for division; and it being determined to try the right eye first, Mr. M'Lauchlan, at seven different sittings, performed the operation. At each successive sitting the cataract was softer than at the preceding one. On the 12th April she was dismissed with the cataract of that eye quite removed, but with the vision impaired, owing to a degree of amaurosis.

*Remarks.* The history of the case indicated a complication of amaurosis with the cataract, and no great hope of a perfect restoration of vision was held out. The result, however, surpassed our anticipations; the patient is now able to direct her own steps, use the needle, distinguish colours, and read a little of a large type. She has again returned, and is now submitting to the same mode of treatment for the left eye. The right eye was first selected for treatment in consequence of the softer appearance of the cataract, and because its vision was barely sufficient to distinguish the shadow of passing objects. No unusual symptoms followed the use of the needle, but the process of absorption was slow.

### Case of Lacerated Wound from the bursting of a Firelock.

W. W., æt. 27, a gardener, admitted at 4, p.m., 24th November, 1836. Twenty-four hours before admission he received a wound of the left hand by the bursting of a firelock which he was holding. The shot and pieces of the gun passed through the hand, carried off the ring and little fingers, and otherwise shattered the hand very much. The wounds had been stitched up immediately after the accident by two respectable surgeons, one of whom accompanied him from his home, a distance of eight miles, to the hospital, and stated that all the metacarpal bones were fractured, that a portion of one had been removed, and that the hæmorrhage had not been great.

*Symptoms at admission.* Hand swelled—tension of the ligatures, and a slight oozing of venous blood. The thumb and two remaining fingers felt cold, and had very little sensation; pain was acute in the thumb and palm of the hand. Tongue coated and moist—skin cool—thirst moderate—pulse 88. The stitches were removed, and warm fomentations applied. From the time of his admission until the 2d December, the hand and arm inflamed, and the wounds of the former suppurated and sloughs formed. During that period warm fomentations and poultices made with the solution of the chloride of soda were applied; the pain was alleviated by opiates, and the bowels which were constipated kept open by laxatives and enemata. On the 2d December, when symptoms of tetanus supervened, and the pain of the hand increased, solution of opium was added to the poultices, and he had Calomel, gr. iij. Camph. gr. v. Pulv. opii, gr. 1½, every four hours. On the following day the symptoms of tetanus increased, and he complained especially of pain at the lower end of the sternum, which came on in frequent paroxysms upon the slightest motion. The discharge from the wounds continued copious. The previous treatment was continued, and a great variety of antispasmodics given by the mouth and rectum without effect. On the 4th the paroxysms of pain increased in frequency and severity, and were followed by convulsions; deglutition was almost impossible, and attempts to swallow renewed the spasms; the skin was clammy; pulse 102, full and regular. Suppuration was still abundant. At mid-day, after a consultation, Mr. M. Lauchlan removed the limb at the insertion of the pronator radii teres by the flap-mode, and a large opiate was exhibited. During the first four hours after the operation the spasms continued, and then ceased. At 6, p.m., however, he complained of a distressing feeling of choking from the presence of viscid matter in the larynx, which he could not discharge. Skin was clammy—pulse 120 and varied. On the 5th, at 10, a. m., the convulsions returned with great violence, and he died immediately; the choking sensation continuing to annoy him to the last. A post-mortem examination could not be obtained. On examination of the detached hand, the whole of the metacarpal bones were found comminuted.

*Remarks.* As not unfrequently happens in such cases, constipation preceded the tetanic symptoms; and the pain at the bottom of the sternum was much complained of; but, contrary to what generally obtains, the purulent discharge continued abundant. It is proper to mention that, among the antispasmodics used, tobacco and the hydrocyanic acid are not included. It will be observed that amputation was delayed until the third day after the appearance of the tetanic symptoms, when all hope of relief from mercury and the various antispasmodics was gone. The operation seemed to produce temporary abatement of the symptoms, but their return in a few hours quickly destroyed the patient. The result of the amputation in this and other cases would seem to point out the propriety of having recourse to it on the first accession of the symptoms or not at all.

The fatal case of hernia humoralis was owing to an attack of typhus fever.

The cases of hydrocele were treated by acupuncturation, but in only one of them did it prove successful. In a case, however, which occurred subsequent to the period embraced in the report, in which it was tried, the fluid was speedily removed. In both cases the hydrocele was punctured several times, but only one puncture was made each time.

The cases of chronic inflammation of the mucous membrane of the bladder were treated with cubebs, inf. buchu, uva ursi, nitric acid, and decoction of pareira brava. Of these the last has been found the most effectual in reducing the secretion of ropy mucus, the pain and frequent calls to void the urine.

The cases of favus were treated with a great variety of remedies, such as the following:—℞. Subcarb. sodæ, ʒiij. Slaked lime, ʒij. Lard, ℥ij. Mixed. ℞. Carb. potassæ, ʒij. Axung. ℥j. Mixed. ℞. Pul. helleb. alb. Pul. conii macul. āā ℥ss. Axung. ℥iv. Mixed. ℞. Liq. potassæ, ℥j. Ol. oliv. ℥ij. Mixed. Nit. argent. Potassa c̄ calce; and, lastly, with mere ablution with warm water twice a day. With all these applications, with the exception of the potassa, we have found the improvement only temporary. It was applied almost daily to portions of the affected cutis, so as to destroy it, and it appears to be the most efficient remedy. This treatment has been tried in three cases, of which two are under treatment. One of them has been so for eight months, without a recurrence of the disease on those parts to which the application had been made. The remedy is a severe one, but as this disease renders a person loathesome both to himself and others, patients for the most part willingly submit to it.

The case of ulcer terminated fatally from an attack of dysentery which supervened on the primary disease.

Several of the skin diseases, and more especially the syphiloid and sibbens, were treated with the solution of the hydriodate of potassa, which we have found very effective in the secondary symptoms of syphilis, and particularly so where there was a scrofulous habit of body. In such cases it has proved more beneficial than mercury and sarsaparilla. We have been forced to intermit it from its producing headache, sore throat, and diarrhœa; and we have witnessed ptyalism and large petechiæ produced by it. The case of eczema inveterata, after being tried alternately with ioduret. ferri, ioduret. sulph., or proto-iod. hydrarg. taken internally, and nit. argent. and sulph. cupri applied externally, was treated with the hydriodate of potassa with little benefit. It was in this case the petechiæ were observed, and it was interesting to see them disappearing and re-appearing as the medicine was omitted or resumed.

---

### III.

### REGIMENTAL HOSPITAL, BANGALORE.

### PURULENT DISCHARGES FROM THE BLADDER AND RECTUM IN HEPATIC DISEASES, &c. By J. MOUAT, Esq.

PURULENT deposits have been noticed from a very remote antiquity, and have been the subject of much discussion in modern times. Instances have been recorded by Galen, Scultetus, Parè, Belloste, Quesnay, Butner, and others, "in which the sudden disappearance of abscesses, and the cessation of purulent discharges, have been accompanied with evacuations of pus by the bladder and rectum.* Yet, so far as our recollection serves, the former has not been noted as a termination of hepatic abscess. Purulent matter passed by stool is by no means a rare occurrence, and has been referred† to the pus escaping by adhesions, and ulcerative communication, directly into the intestines, gall-bladder, ductus-choledochus, &c.; whereas, if our observations be correct, it has in several instances been a deposit or excretion in the fæcal and urinary discharges. Cases, therefore, illustrative of such an event, we trust, will possess sufficient claims to merit the attention of the profession, independent of being characteristic of the beautiful and admirable efforts of the system, in averting the fatal termination of disease in more than one individual, when the resources of art promised scarcely a hope of relief.

We have no intention of entering into a detailed history of purulent discharges or the diseases connected with such phenomena, but to illustrate by a brief abstract of cases, a mode of termination, that would appear, from our own experience, to be of more frequent occurrence than the histories of hepatic affection would lead one to expect. That it should not have been observed and recorded in hepatic abscess, is no reason why it should not have existed. Had it not been accidentally noticed in Ward's case, it is probable it would not have been detected in the subsequent cases,—so prone are we to overlook that which is not expected. The frequency of the occurrence may be referred to the great prevalence of hepatic cases at this station, and their protracted tendency to the excellence of its climate enabling the constitution to make extraordinary efforts towards convalescence, even after the most severe and formidable acute disease. Therefore it is not too much to infer, that in less salubrious places, several of

---

* Dr. Balfour's Essay on Purulent Deposits, 1830.
† Annesley, vol. ii. page 210.

the cases which we have recorded would have terminated fatally, even at a period anterior to the appearance of the pus in the excretions.

Mr. Annesley, in Vol. I., pages 674 and 676 of his elaborate work on the diseases of India, relates a case of hepatic abscess discharged through the lungs, and in whom "a wound, which he received in his thigh, broke out and discharged a quantity of pus with small stones;" and regarding which Mr. Annesley observes; "probably the irritation and discharge from the parts acted as a derivative from the seat of the disease in the liver and lungs." Likewise purulent accumulations take place after wounds of the veins, after fractures, amputations, excision of the joints, wounds of the head, delivery, &c., and consequently from analogy, it is not unreasonable to expect a like occurrence in hepatic affections, since the very actions of the system which could cause the removal of an abscess or its subsequent deposition in a remote and different texture, would occasionally discharge it with the excretions of the intestines and kidneys, had we not the authority of facts for its confirmation.

Hunter declared the translation of pus impossible, and denied that its absorption was attended with pernicious effects. However, Parè, Pegrai, Bonnet, Morgagni, Petit, Van Swieten, Rochoux, Marechal, Reynaud, Legallois, Lary, Velpeau, Mr. Rose, Hennen, Dr. Copeland,* &c., who have directed their attention to these anomalous formations of abscesses in distant situations, have not only admitted the occurrence, but offered various explanations of the circumstance. Where opinions are various and discrepant, it is probable that many of the cases from whence they have originated, have been alike varied and modified. We should, so far as our observations extend, refer the appearance of purulent matter in the fæcal and urinary discharges to absorption and subsequent excretion. Neither can we altogether dismiss the influence of the known efforts of the system in relieving itself when any of its principal functions are oppressed or impeded in their natural actions, as is illustrated by the results of critical discharges, and the phenomena of metastasis, which, however explained, must be allowed to exist; since the most minute dissection could trace no direct communication between the liver and the intestines and kidneys in those who died; nor in Leamy, case 12, between the stomach, the seat of his disease with the lungs and kidneys, though he expectorated pus, as well as voided it with his urine. Beside which, Twining observes, that small abscesses near the surface of the liver, are frequently absorbed, and that in two cases of hepatic abscess, he "found a small quantity of puriform matter in the right ventricle of the heart."† Andral and others have observed pus in the veins, and Dr. Alison states: "The veins leading from extensive collections of matter, especially in chronic cases, are often found loaded with pus."‡ We may, therefore, look to the vascular system as the more probable mode of its removal and subsequent excretion. After all, how this takes place is of little moment to inquire, and perhaps in some degree is irrelevant to the object of the present communication, our wish being merely to point out the circumstance, and to give a brief record of the cases illustrative of purulent deposits in the hepatic diseases of this country. Such an occurrence being established, it is easy to raise a theoretical superstructure; but though we may be unable to trace with more than speculative precision the operations of Nature by which so important a result has so frequently given unexpected relief, yet none, we imagine, can help admiring the extreme beauty and importance of the effect, as illustrated by some of the following cases.

---

* Vide Dr. Balfour's Essay.

† Diseases of Bengal, page 140.

‡ Vide also Graves on the Lymphatic System.

*Case* 1.—Corporal James Ward, ætat. 26. In India 1 year.

Admitted on the 29th September, 1831, with a severe attack of hepatitis, attended with giddiness, fever, and slight cough. Pulse full and frequent; tongue white; bowels loose, and stools mixed with slime and blood. Though actively treated and copiously bled, yet twenty-seven days after admission his right side was observed enlarged, and which continued to increase gradually till the 23rd November, or fifty-seven days after admission, and whilst waiting anxiously for the abscess to point, it was observed that he passed matter by stool—and on the 8th December, large quantities by urine, so that by the 22d January, 1832, the swelling had quite subsided. He, however, remained in hospital many months, and though his general health was good, yet his side occasionally became painful and enlarged, which was always relieved by his passing matter either by stool or urine. The whole side at times appearing enlarged with evident fluctuation, yet as it did not appear to point, it was deemed uncertain and hazardous to operate. He remained much in the same state till December, 1832, when he was invalided and sent to Púnámállí, and ultimately to England. Late accounts report his perfect recovery.

In this case the cure was effected by the efforts of the constitution, and the passing the abscess by stool and urine, but principally by the latter secretion. There were no indications that the abscess had burst into the intestines; and its gradual removal, and that also by urine, would discountenance the supposition.

*Case* 2.—Private Thomas Rippen, ætat. 21. In India 2 years.

Admitted on the 8th February, 1832, with acute hepatitis—viz. severe pain in the right side extending to the shoulder, much increased on full inspiration and on pressure. Pulse quick and full: tongue white and moist: bowels costive, and skin hot. Though considerably relieved by active treatment, yet the pain of side constantly returned, with fever, functional derangement, &c. till the 27th April, 1832, or eighty days after admission into hospital, when pus was observed in his stools, and which he continued to pass in large quantities for twenty-seven days, with evident relief to his side. He remained delicate, sallow, and weak for some time, but ultimately got quite well, and on the 30th June following was discharged to his duties. During his sickness the side was not observed enlarged. He had a relapse, and was re-admitted into hospital on the 11th October following with a similar attack; his side was now obviously enlarged, and as he did not improve, and his constitution giving way, he was invalided and sent to Púnámállí on the 5th December, 1832, for a change of air, where he died in the depôt hospital, the 9th February, 1833.

The subsequent history of this case is equally interesting. On his arrival at Púnámállí 25th December, his right side was enlarged, and he had copious expectoration of purulent matter. On the 30th, left leg swollen from the thigh to the foot, which came on during the night. 31st, considerable swelling of lower extremities. January 2nd, swelling very painful.—4th, stools contain matter and blood.—14th, incision of right side between the ribs and a quantity of matter discharged.—22d, swelling of left leg decreasing, but right increased. February 9th, expired. No dissection recorded, but it would appear by the testimony of Assistant Apothecary Peppin, who was at that time at Púnámállí and assisted at the autopsy, that there was adhesion between the liver and diaphragm, and between the latter and the right lung, but no communication with the intestines, or any disease of this canal. It would appear by the case itself, with which we have been kindly furnished by our friend Dr. Stephenson, that he expectorated pus till about the time the incision was made in the side, but that by the intestines, continued to the period of his death.

This case is particularly interesting,—

1st. From the cure having been effected in the first attack by the abscess passing by stool.

2nd. Upon its re-appearance, the discharge of purulent matter by the lungs, in consequence of ulceration and direct communication through the diaphragm, as well as by the intestines and by the incision from the abscess itself.

3rd. The great constitutional irritation, the inflammatory swelling of the legs, so analogous to some of our own cases, and therefore how much is it to be regretted that the history is so defective, and that the pathology was not recorded by the medical officer then in charge.

*Case* 3.—Private Joseph Gibson, ætat. 23. In India 2½ years.

Admitted on the 5th April, 1832, with a violent attack of hepatitis, attended with sharp pain in epigastrium, extending to the right side and shoulder, with short dry cough and febrile symptoms, &c. Was largely bled and actively treated: yet on the 20th day after his admission, he spat up, by coughing, large quantities of purulent matter, tinged with bile, and of a bitter taste, with great relief to the dyspnœa, pain, cough, &c. Ten days after this he passed matter by stool and also by urine, which for a time relieved the pain of his side. However, he became hectic, wasted rapidly, and died on the 14th May, about six weeks after admission.

On *dissection* two abscesses were found in the right lobe of the liver: one adhering to the diaphragm and communicating with the lung, but small and nearly healed; the other was large and without any communication with the intestines, biliary ducts, or kidneys, notwithstanding the most minute examination.

Here were extraordinary efforts made by the powers of the system. That by the lungs had nearly succeeded, but the constitution sank ere the second abscess could be removed by the stools and urine.

*Case* 4.—Private James Young, ætat. 27. In India 6 years.

Admitted on the 14th April, 1832, with hepatitis; complaining of sharp pain in the right side, increased on pressure and deep inspiration, with much constitutional disturbance. Was largely bled and actively treated, but continued sickly, sallow, and debilitated till 13th May, when pus was observed in his stools, which he continued to pass in large quantities, until the 22nd, or for nine days. From this period he began gradually to regain his strength and appetite, and on the 31st July was discharged cured, and has remained since that time at his duties quite well, though looking sallow.

Here nature effected a cure by voiding the abscess by stool. There was no sudden giving way or other circumstance that could lead to the supposition that the abscess had burst into the intestines, &c.

*Case* 5.—Private Robert Mallalew, ætat. 28. In India 4 years.

Of a healthy appearance. Admitted on the 29th June, 1833, with symptoms of acute dysentery, complicated with acute pain at epigastrium, much increased on pressure; though the severity of the bowel-disease was considerably relieved by active treatment, yet he continued to suffer from the pain at epigastrium till the 7th July, when it extended to the right side, attended with fever, slight dry cough, quick pulse, thirst, &c. This continued unabated, though copiously bled and actively treated, till the 23rd August, or fifty-six days after admission, when pus was observed in his stools, and the following day more largely in the urine, with some relief to his suffering. From this period up to the 6th September he continued to pass matter copiously both by urine and stool, but without experiencing permanent relief to his side or producing ptyalism, though calomel and blue pill were freely and largely used. On the 7th September the pain much increased, attended with dyspnœa, cold perspiration, restlessness, &c., and he died the following day.

*Dissection.*—Discovered a large abscess in the right lobe of the liver, containing about 13 ounces of well-digested pus; the left lobe appeared sound, the gallbladder thickened and contained a mixture of pus and bile. No connexion could be traced either with the biliary ducts, kidneys, or intestines.

At first the discharge of pus gave great relief, and though he ultimately fell a sacrifice to constitutional disturbance, still it shows the extraordinary exertions nature made to get rid of the irritating cause, and its importance by its temporary alleviation of the symptoms.

*Case* 6.—Private George Kennedy, ætat. 41. In India 11 years.

Admitted 7th September, 1833, with acute hepatitis, complicated with fever, diarrhœa, general debility, nausea, &c. Copiously bled and actively treated, yet he continued to suffer till the 18th, when his mouth became affected from calomel, and the following day pus was seen in his stools, which he continued to pass largely both by stool and urine for several days, with considerable relief; but his strength rapidly declined with emaciation and general debility. He died on the 27th, or twenty-one days after admission.

*Dissection*.—Discovered two distinct abscesses in the right lobe of the liver, one on the superior surface just below the diaphragm, containing about 19 ounces, and the other on the posterior margin, about 5 ounces of pus: the left lobe appeared sound, the gall-bladder distended with about two ounces of dark-coloured bile. No communication could be traced either with the intestines or kidneys, though minutely examined.

Here again we see the great efforts of the system, its effects, and the consequences of the failure.

*Case* 7.—Serjeant George Munnings, ætat. 43. In India 22 years.

Admitted on the 21st February, 1834, with pain in the right side, extending to the shoulder, increased on pressure and on full inspiration, with febrile symptoms, &c. These symptoms continued for several days, sometimes the pain increasing and at others abating, with frequent attacks of rigors, and swelling and heaviness of right side. Pulse varying from 90 to 134°, soft. On the 27th, or six days after admission, he began passing matter by urine, to the extent of 2 ounces in 24 hours, which considerably relieved the pain of his side, but left a partial swelling and heaviness; he continued to pass matter for several days, until his mouth became affected from calomel, when it gradually decreased, and with it the pain and swelling of the side subsided, but leaving him for some time in a weak state; appears drawn down to one side as if adhesion had taken place from the attack, with absorption of a portion of the liver, as we see in affections of the chest.

Invalided in consequence of debility, and sent to Púnámállí 16th December, 1834.

This was a very interesting case, as the tumour of the side, and the other symptoms, with the passing of purulent matter, plainly indicated its nature. The cure in this case was performed by the effects of the system.

*Case* 8.—Private Michael Redy, ætat. 40. In India 15 years.

Admitted 12th July, 1834, with acute hepatitis, attended with nausea, vomiting, general weakness, loss of appetite, fever, &c. On the following day the side appeared enlarged and very tender on pressure. Like the other cases, was actively treated, yet the pain and swelling continued unabated till the 17th, when his mouth became affected from mercury, and at the same time a deposition of pus took place in the urine, with great relief to the pain, &c. and the swelling appeared somewhat reduced. From this period he continued to pass matter in his urine, to the extent of 2 to 3 ounces in the 24 hours, till the beginning of September, when a gradual decrease of both pain and swelling, and an improvement in his health and strength took place. During September, pus was observed in less quantity, and about 20th November it ceased entirely—and on the

26th of that month he was discharged to his duty quite well, and is now enjoying good health.

This is a most interesting case;—the disease so well marked, and so long protracted;—the large quantities of matter daily passed, and his ultimate recovery. The pus was repeatedly analysed.

*Case* 9.—Private Mark Smith, ætat. 23. In India 1 year.

Admitted 18th June, 1834, with acute hepatitis, complicated with dysentery, slight fever, nausea, general debility, &c.; copiously bled and actively treated, yet on the 5th day of admission his side became much swollen and very painful, and the following day pus was observed in his urine, with evident relief; the swelling was now considerably reduced, and there was slight ptyalism from the mercury he had taken. On the 24th the pain returned with great violence, (though he continued passing matter largely, both by urine and stool,) followed by difficulty of breathing, weakness, copious cold perspiration, dyspnœa and lowness of spirits, &c., and he died on the 26th, or eight days after admission, in great agony.

*Dissection.*—Disclosed several abscesses of various sizes in both lobes of the liver, some running into each other, and others quite distinct and filled with pus, but no communication could be traced either with the kidneys or intestines,—though the urinary bladder appeared filled with a mixture of urine and purulent matter.

Here again were the efforts of the system powerfully called into action, but the disease was too extensive, and he sunk in the contest.

*Case* 10. Private John Thomas, ætat. 26. In India 4 years.

Admitted 27th October, 1834, with symptoms of dysentery and much fever. Was bled and actively treated, and he appeared much relieved. About the beginning of November he suddenly experienced pain in the region of the liver, attended with rigors, cough, dyspnœa, fever, &c. Again copiously bled, &c., but he continued to suffer till the 12th, when his mouth became affected from the mercury, and the following day pus was observed in his urine with considerable relief. On the 13th the side appeared enlarged, and the matter in the urine was increased. From this date he continued to pass matter by urine daily, to the extent of from 1 to 3½ ounces in the 24 hours, till the 24th, when it began to decrease, and the side to enlarge and become more painful, the swelling extending across the epigastrium. His suffering now became so great, that an attempt was made on the 28th to open the abscess by cutting down on the peritoneum with hopes of exciting an union with the external parietes of the abscess; but fortunately it was not punctured, for at that part (as was afterwards observed) there was no adhesion. He continued suffering severely till the 14th December, when he suddenly expired.

*Dissection* shewed an enormous abscess in the right lobe of the liver, which had destroyed the parenchyma, leaving a large cavity containing not less than 6½ pints of well-digested matter. No communication could be traced with the kidneys: the bladder contained about 2 ounces of urine and pus. The abdominal cavity was full of serum and purulent matter mixed together.

Great as had been the efforts of nature in this case, yet the ravages of the disease were too extensive to admit of relief. It is only astonishing so much was performed.

*Case* 11. Private Henry Jackson, ætat. 32. In India 8 years.

At one period stout and strong, but at present sallow and looks delicate. Has suffered for some time from slight hepatic disease, complicated with slimy, mucous stools and dyspepsia. Was taken into hospital on the 30th November, 1834, when he had slight pain in his right side extending to the shoulder, soreness across the abdomen, loss of appetite, and general weakness : quick soft pulse and tongue, but no fever. Was actively treated and bled with partial relief to the pain of side, leaving merely a soreness in the region of the liver, with occasional headache, flatulence and debility. On the 5th January he complained of palpitation of the heart without pain, but on the following day the pain returned for two days in the right side extending to the epigastrium and left side : was much relieved by leeches, fomentations, &c. On the 10th of the same month his mouth became affected : ptyalism was copious, though pain and soreness continued more or less till the 14th, when pus was observed in his urine and afterwards in the stools, with evident relief to his suffering. On the 16th the pain and soreness again became violent, though he still continued to pass matter, but in smaller quantity :—after this the pain and soreness shifted from place to place, sometimes in the left side, sometimes in both shoulders or epigastrium and abdomen, with severe headaches, occasionally attended with returns of the palpitation, lassitude, cold sweats, dyspepsia, &c., with increasing emaciation and hectic symptoms. The matter passed by stool gradually decreased till the fifth February following, when it entirely ceased. After which he suffered comparatively little till the 10th April, when a marked change took place, and he got rapidly better, and on the 16th was free from complaint. On the 26th discharged quite well. Continues sallow, with pain at times in his side or belly, and dyspeptic, with griping or mucous stools occasionally.

This case is remarkable,—

1st. Passing pus by urine, and then by stool, and these with evident relief to his maladies.

2nd. The great constitutional disturbance and violent irritation from time to time, as well as the protracted convalescence, all indicating hepatic abscess, and its removal or excretion by the urine and by stool.

*Case* 12. Private Michael Leamy, ætat. 29. In India 6 years.

Delicate appearance, and sallow complexion. Has suffered much from an attack of splenitis, and subsequently, swelling of both feet. Admitted into hospital on the morning of the 26th April, complaining of pain at the epigastrium and in the left side, in the situation of the spleen, slight swelling of both feet, and great debility. The swelling pitting on pressure. He was relieved by leeches and fomentations; but the following day he complained of pain in the right groin, along the course of the vessels, unattended by hardness or swelling. This was also relieved by leeches. On the 2nd May he again complained of pain in the epigastrium, accompanied with strong palpitation, also of pain in the course of the femoral vessels of both thighs, and about 9 P. M. vomited a large quantity of purulent matter, and the following day pus was observed in his urine. He continued to vomit it occasionally, but in smaller quantities. On the 5th he passed some pus by stool, but had ceased to vomit any ;—still had pain at the epigastrium and palpitation. On the 13th he began to cough, and on the 16th the cough became more severe, with expectoration of purulent matter. He continued to pass pus by urine, by expectoration, and occasionally by stool, until the 23rd, when his legs became more swollen with pain in the course of the femoral vessels. This was relieved as before, but the swelling continued slowly to increase, and on the 7th June he complained that his limbs felt dead, and that he tottered when walking. On the 4th July the left leg and thigh became tense, elastic and swollen, and he had pain along the saphena and femoral veins, which felt distended and cord-like. Leeches were applied with relief, but the hands now began to swell; and on the 11th the distention and pain of the vessels returned, extending as far as the ankle; the pain was again relieved by leeches, but the vessels continued distended and cord-like, with swelling and numbness of both hands and feet, and occasional palpitation. He again improved a little, the swelling of both hands and feet nearly disappearing, his cough ceasing, and pus only occasionally seen in his urine. However, on the 21st, his belly was observed tumid, with evident fluctuation, and he had passed no urine for three days. Next day he made a small quantity which contained pus, and the belly became rather less. He continued in this state up to the 27th, when the left leg and thigh again became swollen, shining and elastic, the belly more distended, and the anasarca extending to the loins. He looked low and anxious, urine scanty and high-colored, still containing pus. On the 29th the left leg from the groin to the foot was much swollen, tense, and generally painful, and in some parts of a violet color, and felt cold. About noon he began to sink, and at 1 o'clock expired.

*Sectio Cadaveris at* 5 P. M. *four hours after death, by Doctor Clark and Mr. Trail.*

Great emaciation, abdomen tumefied, and left leg considerably swollen.

*Head*—Brain healthy.

*Thorax*—All the viscera small, but healthy: some fluid in the cavity of the thorax. No adhesion between the diaphragm and lungs.

*Abdomen*—Liver particularly small, apparently free from disease. Stomach small and contracted, with thickening of its coats, but no adhesion to diaphragm: cavity full of a muco-purulent fluid: scirrhous enlargement of the pyloric portion of the stomach: the parts externally had a cartilaginous feeling, and on cutting into them the coats were found thickened to the extent of about half an inch: the parts internally were extensively ulcerated, and several large fungoid carcinomatous-looking tubercles sprouting from the mucous membrane, and nearly closing up the pyloric orifice. Spleen wrinkled, and had something like a cicatrix on its upper surface. Intestines contracted and of a very pale color. Other viscera healthy: seven pints and a half of extremely limpid fluid in the cavity of the abdomen.

Extensive disease in the veins of the left inferior extremity. The diseased state commenced just at the formation of the vena cava, but did not affect that vessel:—the iliac of the right side was affected for about an inch, but no more—the left iliac, external and internal: the femoral and all the branches were full of clotted and nearly organised blood, so as to render them almost impervious: this diseased state extended about half-way down the leg:—limb full of fluid, and a good deal of fatty matter in the sheath of the vessels; and on the thighs, considering the generally emaciated state of the subject.

This case is very interesting; as much from the diversity of symptoms, as the extent and variety of parts diseased, the pathological appearances, and their connexion with the diseased phenomena observed during life. It was regarded with much interest by most of the medical men at this station, and is remarkable as to,

1st. The vomiting of pus.
2nd. The pus observed in the urine.
3rd. The passing of pus by stool.
4th. The expectoration of pus.
5th. The swelling and disease of the veins of the lower extremities.
6th. The disease having the pathognomonic symptoms of beri-beri, and phlegmasia dolens or phlebitis in the male.
7th. The malady originating from scirrhus of the stomach, and therefore it is to be inferred, the pus expectorated and passed by urine must have got into the circulation from thence; since,
8th. There was no communication between the stomach and diaphragm, or diaphragm and lungs, or any traces of disease in the thorax.

The diseased state of the veins, and the attendant symptoms so illustrative of the three cases already sent to the Society, and the first of which was published in part 2, Vol. 7 of the Medical and Physical Transactions.*

---

* Vide Appendix, A.

*Case* 13. Private Charles Pearson, ætat. 21. In India two years.

Stout make, and pale, and leuco-phlegmatic appearance.—Admitted into hospital on the 21st August, 1834, with symptoms of acute dysentery. Partially relieved, but had relapses on the 4th and 18th September, 9th and 26th October, and again 7th November; and on the 21st November, or ninety-two days after admission, felt acute pain in the right side, extending to the shoulder. On the 23rd, pain in the right thigh in the course of the femoral vessels to the ankle, but this was relieved by leeches, fomentations, &c. On the 27th, again had pain in his right side and hip, but was relieved by leeches, and he continued easy and mending up to the 28th December, when he had some return of the dysentery. On the 1st January, 1835, had palpitation of the heart; and on the 5th, pain in chest and dyspnœa, and during the night slight delirium; and a feeling of sinking on the 6th, with frequent dark stools. On the 9th was again suddenly attacked with pain in right-thigh and top of the right shoulder, the femoral vessels felt hard and cord-like, with pain extending down the thigh and tibia: leeches applied. 10th, pain and dyspnœa much relieved. 12th, cough and dyspnœa when lying down. 15th, pain now in both legs along the course of the saphena veins, and they are swollen and tense, though relieved by leeches. 16th, feet observed to swell and continued swelling, but with less pain, till the 31st, when pus was observed in his stools. Feb. 2nd, pain now returned in the right-leg in the course of the femoral vessels with hardness: relieved by leeches and fomentations. 5th, pain from the tip of the right-shoulder extending down the side to the groin, and thence to the instep; femoral vessels hard and distended, with strong palpitation, also pulsation at the epigastrium and both iliac regions. 11th, severe pain again in the left-leg from the groin to the ankle, with swelling and hardness of the femoral vessels, also pain in right-arm from the deltoid muscle to the elbow; relieved again by leeches and fomentations: pus still passed by stool, and he is weak and appears sunk, and vomits every thing. 14th, much relieved. 15th, pain returned in the course of the iliac vessels of the left-leg and relieved by leeches. 16th, feet less swollen. 20th, left knee contracted, less pus in stools. 23rd, pain returned in the left-leg and foot; saphena enlarged and painful, also the femoral veins. 25th, bursting sensation in the left-foot. March 1st, pain still in the left-foot, with much swelling, tense and elastic; both legs feel dead, numb, and powerless: pus still in the stools. 5th, swelling of the right-leg diminishing, both knees contracted, strong pulsation in the right groin: relieved by leeches. 26th, much relieved; feet swell only whilst walking or standing: pus still in stools, but less in quantity. April 15th, pain in right iliac region, extending down to the knee: femoral vessels again hard and sore on pressure, also soreness in the saphena of the left-leg: relieved again by leeches and fomentations: no appearance of pus in stools since 1st April. May 5th, pain again in right shoulder, side, and groin, extending down to the inner ankle: relieved as usual by leeches, fomentations, and hip-bath. 6th, a good deal better. 12th, a return of pain in the right-side and epigastrium, but relieved by leeches. 13th, much better, and appears easy; slight numbness and bursting feel in both feet. 20th, mending, though slowly. June 12th, left leg and foot again swollen, and pit on pressure; the saphena distended and painful from the middle of the thigh to the ankle: relieved as before by leeches and fomentation. 16th, numbness in the left instep; the veins of both legs blue and much distended; palpitation on the slightest exertion: bowels regular. August 19th, bled; and the 24th, the vein became inflamed and ulcerated, pain and hardness extending up the arm, with hardness and stiffness, &c. 27th, saphena of the right-leg enlarged, painful, and full of knots: much relieved by leeches and fomentation. 29th, relieved and doing well: only slight return of palpitation and numbness of both feet. August 1st, some pains in both hips, knees and insteps: sixteen ounces of blood abstracted from the arm gave him relief. 13th, pains left him. 26th, veins of both legs swollen from above the knees down to the ankles, and pain on pressure; also a tingling sensation on the outside of the left foot. 28th, pain and swelling of the veins left him. September 12th, return of pain in the left-foot, with heavy burning feel; veins not affected: a few leeches and fomentations gave him relief. 13th, pain gone, and succeeded by numbness. 29th, return of cough and dyspnœa and pains in all his joints. October 3rd, somewhat relieved by warm-baths and the usual expectorants. 18th, some pain again in both legs, and the veins are swollen; still cough and dyspnœa, with copious expectoration of phlegm mixed with pus: the affection of the limbs much relieved by leeches and fomentation. 21st, pain again on inside of both legs, right-side, and shoulder; the veins of the legs slightly swollen and painful: was bled to twelve ounces, and had some leeches applied with relief. 22nd, cough continued, with copious expectoration of pus; much pain in femoral and saphena veins of both legs: obtained relief by leeches and fomentation. 23rd, cough much relieved; free expectoration of pus, which had been tested by sulphuric acid:—in the evening severe pain in the right groin, with thickening and hardness of the crural veins, and strong pulsation in them: relieved a good deal by leeches. 24th, much easier, less hardness of vessels. 25th, feels weak and low, less cough, and still expectorates pus. 26th, pus observed in urine; pain and hardness of the vessels entirely left him; but in the evening felt a shooting pain in femoral vessels of both legs, and had leeches applied with some relief: much pus in urine. 27th, feels poorly, less pus in urine, veins of the right-thigh greatly enlarged, sore and painful: leeches applied with relief. 28th, veins soft and more elastic: less pus both in urine and expectoration. November 1st, severe return of pain again in the right-side and shoulder: increase of pus in urine: veins of both legs swollen. 2nd, had an attack of epilepsy, appeared delirious and insensible, and convulsed foaming at the mouth: pupils dilated: these symptoms were much relieved by leeches and a purgative enema, and in two hours was sensible and quiet. 3rd, slept well, and felt easy: much pus in urine. 4th, much cough with expectoration: a good deal of pus in urine. 5th, pains in calves of both legs; less cough and expectoration; increase of pus in urine; some palpitation: pus in small quantity observed again in stools.

This man is still in hospital, and under treatment, and the prognosis must consequently be uncertain, if not unfavourable, though the swelling is gone down, and he now walks about.

This case, however, is not only connected with the object of the present paper, but presents many features of great interest in its varying symptoms, and the textures assailed.

1st. The frequent relapses of dysentery, and these terminating,

2nd. In hepatic disease, and the frequent return of pain in the side: and this alternating,

3rd. With frequent attacks of inflammation, &c. in the veins of the lower extremities.

4th. The phlebitis after venæ sectio, indicating both the morbid state of the habit, and the inflammatory disposition of the venous system.

5th. The appearance of pus in the stools.

6th. The purulent matter expectorated.

7th. The pus passed by the urine with the invariable effect of relieving or quieting the constitutional disturbance.

8th. It becomes a question, whether the pus voided was from hepatic abscess, or venous inflammation, or both?

9th. The œdema, the anasarcous tendency, the painful, tense, elastic swelling of legs and feet, the shooting pains, the contraction of the legs, the dead feel, or numbness in the feet, the tottering gait, the cough, the dyspnœa and affection of the chest, the palpitation, epigastric pulsation, and cardiac affection, the expectoration, the disease of the veins, the anxiety, the peculiar look of distress, &c., so indicative of violent constitutional disturbance, as well as being the pathognomonic symptoms of beri-beri, venous inflammation, and phlegmasia dolens *.

10th. Is it too much to assume, that this man's life has been hitherto protracted by these discharges or excretions by the intestines, bladder, and expectoration; and that had it not been by the relief they afforded, he must have sunk from constitutional irritation and disturbance?

In looking over the thirteen cases, just detailed, it will appear, six have been fatal; six have recovered, though one of these had a second attack, which carried him off; and that one is still under treatment. Of the foregoing, eleven passed purulent matter by urine; and eight of these likewise by stool; and four of them pus by expectoration. But this will be more evident and better illustrated by the following table as one only discharged pus by the rectum, and three others solely by the bladder.

*Table of Purulent Discharges.*

| Number. | Names. | Pus Passed by | | | | Remarks. |
|---|---|---|---|---|---|---|
| | | Urine. | Stools. | Expectoration. | Vomiting. | |
| 1 | J. Ward ...... | 1 | 1 | 0 | 0 | Recovered. |
| 2 | T. Rippen .... | 0 | 1 | 1 | 0 | Do., but died the 2nd attack. |
| 3 | J. Gibson .... | 1 | 1 | 1 | 0 | Died. |
| 4 | J. Young ...... | 0 | 1 | 0 | 0 | Recovered. |
| 5 | R. Mallalew.... | 1 | 1 | 0 | 0 | Died. |
| 6 | G. Kennedy.... | 1 | 1 | 0 | 0 | Ditto. |
| 7 | G. Munnings .. | 1 | 0 | 0 | 0 | Recovered. |
| 8 | M. Reddy...... | 1 | 0 | 0 | 0 | Ditto. |
| 9 | M. Smith...... | 1 | 1 | 0 | 0 | Died. |
| 10 | J. Thomas .... | 1 | 0 | 0 | 0 | Ditto. |
| 11 | H. Jackson .... | 1 | 1 | 0 | 0 | Recovered. |
| 12 | M. Leamy .... | 1 | 1 | 1 | 1 | Died. |
| 13 | C. Pearson .... | 1 | 1 | 1 | 0 | Under treatment. |
| | Total.... | 11 | 10 | 4 | 1 | |

We shall add little to the foregoing cases, except that the deposits by stool, urine, and expectoration were examined in all the patients in the usual manner by the most approved tests, particularly the sulphuric acid, &c.; and though these be of a negative nature, still when taken together with the appearance of the matter voided, left no doubt of its nature.† Beside which, the previous symptoms in some, the swelling of the side in others, and the dissection in all the fatal terminations, are confirmative of the circumstance. One or two cases, selected to illustrate particular views, might create doubt, but there can be none where there were so many instances, many of them protracted, all closely watched by the other medical officers of the corps, and many of them viewed with great interest by professional friends; and where the dissections in the fatal cases were made by the assistants attached to the regiment, with the specific purpose of tracing, if possible, any direct channel of communication between those abscesses and the intestines, lungs, and kidneys. It is true, that in Case No. 5 there was a mixture of pus and bile in the gall-bladder, and no clue as

* Vide Appendix, A and B. † Vide Appendix, B.

to the channel by which it got there; yet even in this case there was no direct communication by which to account for the purulent deposition in the urine.

May not the lateritious sediment observed by practical authors in some acute cases be of this nature? It is only of late years that animal chemistry* or pathological inquiry can be relied on. At any rate, has it not in several of our cases appeared critical,—in all affording more or less relief, and in some cases simultaneous with the affection of the mercury on the system? We are aware that this is not consonant with generally received opinions, or with Mr. Annesley's views in vol. 1, page 528, on the diseases of India. But we have seen too many cases of extensive disorganization in dysentery and hepatitis with death, notwithstanding the most free action of mercury. Still, as a general rule, it certainly holds, that the remission of acute disease is generally synchronous with ptyalism, and its absence indicative of ulceration, and an unfavourable termination; and our experience would shew that the appearance of pus always tended to quiet the constitutional disturbance, and alleviate urgent symptoms; and though temporary in several of the cases, still its powerful influence and great importance needs neither further illustration nor more direct proof, and, if we may draw conclusions from the foregoing cases, it is by no means a rare event, and therefore the observations of others, it is anticipated, will confirm the accuracy of our statements.

J. MOUAT, M. D.

*Bangalore, December,* 1835. *Surgeon, H. M.* 13*th Dragoons.*

---

### APPENDIX.

A.

We are aware that phlegmasia dolens and phlebitis in puerperal females are still, by some, considered distinct and separate maladies.

Phlegmasia dolens is said to be of a rare occurrence and seldom fatal: phlebitis frequent and very dangerous. The swelling in the former *tense* and *elastic*, the parts quickly rising, and the fluid not to be drawn off by puncture: the swelling in the latter *pitting* and *œdematous.* The pathology of the one unknown, the other ascertained by multiplied dissections. Our cases have presented the swelling tense, elastic, shining, pitting on pressure, and œdematous in the same patient during the varied stages of the same attack, and which leads us to consider them different degrees or modifications of the same affection, both as to violence and the order of vessels and tissues implicated, as well as some peculiarity of action in the vessels themselves, as is seen after the application of blisters, where, though the discharge is generally watery and limpid, yet in some rare cases it becomes a gelatinous mass.

A minute analysis of the various histories of Beri-beri would be both interesting and important. It would, at any rate, promote inquiry, correct errors, and tend to elucidate its phenomena, and probably reconcile many of the apparent anomalies in its descriptions, as well as fix its connexion with other diseases; also desirable, as defining its nature when occurring as an idiopathic or acute affection, as well as its appearance in its more chronic and protracted forms, either as a sequela to, or conjoined with morbid states of the system: as for instance, at Rangoon in 1824, when prevalent in Sir A. Campbell's army, and complicated with a scorbutic habit.

---

* Dr. Prout's analysis is the only one we have seen of critical deposits, and it is very possible it may not include every variety of these discharges.

B.

Mr. Mac Grigor, of H. M. 39th Regiment, has kindly favored me with the following communication on the analysis of Pearson's urine.

"In undertaking at your request the examination of the urinary deposits which were supposed to contain pus, I was perfectly conscious of the difficulty of the attempt, both from the want of habitual practice in nice chemical manipulations, and also from the imperfect means and apparatus which I could procure. My examination, therefore, can only be regarded as *tentative*, and as furnishing only an approximation to the truth.

The first specimens with which I was favored resembled much the appearance of pus diffused in water. But as the urine which contained the deposit was highly ammoniacal, I was led to suspect that some of the earthy phosphates entered into the composition of the deposits. From the amorphous appearance I suspected phosphate of lime. Latterly I obtained some fresh specimens of Pearson's urine, and in them no deposit took place till partial decomposition of the urea commenced.

On specimens of the earlier deposits I made some trials of the effect of sulphuric acid strong and weak, of nitric and muriatic acids. With strong sulphuric acid the deposit became red and then black when heated, but a great part remained undissolved in the bottom of the tube. With stong nitric acid, slightly diluted, a yellow tinge was communicated to the mass, with partial solution. When the soluble part was separated from the insoluble part, and slightly neutralized and tested with oxalate of potash, a white precipitate subsided, which readily dissolved in nitric acid; a similar precipitate was obtained by oxalate of ammonia, in the solution obtained from the deposit when treated with muriatic acid. By collecting the deposits which subsided in the urine of several days, about ℈v. of the mass was obtained. When heated with diluted nitric acid and digested for some time and filtered, to separate the insoluble from the soluble part, the last substance was tested with a solution of silver; no change took place till the solution was neutralized by ammonia. When this took place, a mixed yellow and white bulky precipitate formed, which was soluble in an excess of ammonia, and also in an excess of nitric acid. When exposed to the light it soon blackened. This precipitate answers to the white perphosphate and yellow phosphate of silver, which becomes the blackened sub-phosphate when exposed to the light. The solutions then showed the presence of phosphoric acid and lime. The insoluble part washed, dried and weighed, amounted to grains x. showing a loss of ℈ivss. which was dissolved by the nitric acid. This insoluble part was yellow when moist, but became by drying of a reddish brown color. This was again triturated and digested with carbonate of potash, (to make use of Grasmeger's test) a part of the powder remained undissolved, but the solution became of an orange red color, which communicated a yellow tinge to the lime filter—in fact, had some resemblance to carbazotate of potash. This will require further examination.

The difficulty of determining the nature of the animal matter is very great, on account of the want of a proper test to distinguish pus when mixed with other substances. Sulphuric acid is mentioned as a solvent of pus by almost all chemical authors on the authority of Darwin. But Andral has lately made experiments which give another action to strong sulphuric acid. He found that all specimens of pus are invariably first reddened, then blackened by strong sulphuric acid. My experiments confirmed the latter view. Sulphuric acid had exactly the same effect on hepatic pus, on mammary pus, and on the animal matter, which existed in the deposits in Pearson's urine. Dr. Hope found, that albumen itself behaves in a similar manner, and fibrine of the blood is mentioned by chemists to have the same action under strong sulphuric acid. I treated some decolorized clot with sulphuric acid, and found the same action,—redness followed by blackening—to occur. Nitric acid converts pus into a yellow flocculent mass, and communicates the same yellow color to the solution, and closely resembles the carbazotic acid procured by the action of nitric acid on indigo. The animal matter of the deposit had the same color. But then nitric acid renders the skin and other animal textures yellow.

In the course of the comparative experiments on pus, I found that some pus obtained from the mamma of a native woman (who is now six months pregnant, and who had a child eighteen months ago) when dropped into several boiling solutions, coagulated. When dropped into water only a slight appearance of coagulation occurred; in nitric acid a partial coagulum for a short while remained, but afterwards broke into a yellow powdery-looking substance; in boiling carbonate of potash and ammonia a perfect coagulum was obtained; in boiling muriate of ammonia a very consistent coagulum took place even in a very watery specimen of pus.

From most of these experiments, I am inclined to believe that we have no good test for distinguishing pus when mixed with any other substance. I should think that the eye would be the best means of detecting the presence of pus even in urine; taking care, in the first place, to make the examination before an ammoniacal state of the urine caused a precipitate of the phosphates; after which I am inclined to believe that no accurate deduction can be drawn as to the identity of the precipitate with pus. Tests can then only show with confidence the presence of phosphatic portion.

Some blood taken from this patient presented, as to proportion, about an equal division of clot and serum. Blood drawn about a week after to the amount of ℥vj. shewed a great excess of serum, while only a small part of the clot remained. On pouring off the serum in both cases and turning the clot upside down, several decolorized spots were apparent. When this clot was made to float in water, the pale parts freed from hæmatosine became more perceptible. Those white spots ran towards the centre of the clot, but tapering towards their termination. In the clot last obtained some of the pale masses became converted into an excavated scab, and had the appearance of lymph or pus dried. The rounded part in both cases looked like tubercle; or the masses described by Carswell, as appearing in the blood of the spleen of tuberculated subjects."*

---

### IV.

### Calcutta Native Hospital.

Return of Cases of Hydrocele treated at the Native Hospital, Calcutta, by J. R. Martin, Esq. by a retained Injection of Solution of Tincture of Iodine, from 9th March, 1832, to 31st December, 1837.

| | | | | | |
|---|---|---|---|---|---|
| From 9th March to 31st December, | 1832 | .. | .. | .. | 32 Cases. |
| For the year .. .. .. | 1833 | .. | .. | .. | 49 |
| | 1834 | .. | .. | .. | 86 |
| | 1835 | .. | .. | .. | 121 |
| | 1836 | .. | .. | .. | 332 |
| | 1837 | .. | .. | .. | 528 |
| | | | Grand Total | | 1148 Cases. |

---

* This report was read before the Calcutta Medical Society, and printed in their Journal."—*Eds.*

| | |
|---|---|
| Of the above there have been reported failures by the iodine treatment .. .. .. .. .. .. | 3 Cases. |
| Ditto in the experiment of ten cases treated with a retained injection of undiluted port wine .. .. .. | 3 ditto. |
| Cases in which iodine succeeded after the previous failure of the port wine solution and that of sulphate of zinc .. | 9 ditto. |

| | | |
|---|---|---|
| Of the total cases treated there were | Hindoos .. .. .. | 545 Cases. |
| | Mahomedans .. .. | 559 |
| | Christians .. .. | 44 |
| | Total | 1148 Cases. |

N. B. The details of treatment will be found at pages 204 and 411 of the 7th vol. of Medical Transactions of Calcutta, and at page 12 of the Quarterly Journal of the Medical and Physical Society of Calcutta.

---

### V.

### Clinical Contributions from Addenbrooke's Hospital, Cambridge, for the Years 1836—1837. By Henry J. H. Bond, M.D.

In submitting to the notice of the reader the results of clinical observation, derived from hospital practice, the author may be excused perhaps in premising a few words in explanation of the mode in which he conceives such a source may serve to contribute most advantageously to the records of medical experience.

Rare cases, extraordinary in their history, course, symptoms, or results, merit to be reported in such detail as is necessary to convey to the reader an adequate notion of them as distinct facts, and to present them in their individual prominence and peculiarity; and other cases may also be worth narrating fully and historically, where the object proposed is to estimate the value and success of a particular mode of treatment; but ordinary cases, having no such claim to especial notice, might still, it is conceived, *by a condensed report,* be made subservient to the general advancement of medical knowledge in its various branches: and by the cumulative evidence which they might afford in support or in the correction of the current doctrines and alleged facts of the day, compensate for their individual insignificance. They might likewise be made further useful, when derived from various sources, by representing the different phases a disease, familiar in its general features, assumes under different circumstances, as those of condition in life, topography, &c. Indeed it is by such a method chiefly, that the causes of diseases admit of being ascertained, and the assertions and generalizations of authors in their monographs of particular maladies may be submitted to verification.

The matter for consideration then is, how such an appropriation of ordinary experience, of common-place cases, to the elucidation or confirmation of speculative doctrines or practical principles can be best effected? The reduction of the elementary facts of such cases to formal tables would fail probably of accomplishing this purpose, both by the uninviting nature of the materials, and by the want of concentration in the results;—whereas, on the other hand, if all specification of the numerical amount of the facts, from which it is pretended to deduce the inferences, were omitted, the method proposed would have no claim to more consideration, as furnishing *direct evidence* on particular points, than the statements of individual opinions which have been adopted independently of any rigid calculation of data, and which can be accepted only on the credit of the author's sagacity or penetration. It is not meant that this latter mode of deriving principles from the general impressions rather than the rigid deductions of an extensive experience, *may* not be equally and even more valuable—may not embrace and represent larger and more important truths—it is only intended to maintain that the other mode of arriving at conclusions from a rigid analysis of reported cases has its separate usefulness, its peculiar value: the conclusions it affords may be more limited in their application, but they give the reader an opportunity of knowing the extent of the data from which they are derived and of estimating them accordingly—and moreover, such a method may serve as a test, a commentary on the results of the first and ordinary application of experience.

Without pretending to adhere rigidly to any particular plan, it will be the endeavour of the author in the following reports on particular diseases, to keep the above objects in view, and to combine something of the precision of the numerical method with the inductive tact which all practitioners must necessarily employ, in the attempt to render experience subservient to the advance of their professional knowledge.

It may be proper to preface these reports with a short account of the field and opportunities which Addenbrooke's Hospital affords for clinical observation.

The number of beds it contains has lately been increased from seventy-eight to one hundred, and there are seven large and two small wards in the principal building, and two wards in a detached building, mainly intended for febrile disorders, but applicable to the general purposes of the hospital at the discretion of the medical officers. All disorders are admissible; but, with the exception of accidents, each patient is required to have a recommendation from a subscriber. One day in the week is appropriated for the admission of patients, but practically there is no restriction for acute cases or such as require immediate assistance. Out-patients attend once a week. About half the number of patients, out and in-patients combined, are inhabitants of the town of Cambridge—the remaining half come from the county of Cambridgeshire and the Isle of Ely, from the contiguous parts of the adjoining counties of Essex, Hertfordshire, Huntingdonshire, &c. This circle embraces several considerable towns, furnishing a portion of the patients, as Ely, March, Wisbech, Royston, Newmarket.

The medical officers consist of three physicians, three surgeons, and a resident apothecary. Post-mortem examinations of patients dying in the hospital are rarely omitted, and not unfrequently opportunities occur for necroscopical enquiries among the patients dying at their own homes. No inconsiderable proportion of the morbid preparations of the extensive museum of the University is derived from this source. When practicable, notices of the operations to be performed are circulated among the practitioners of the town and neighbourhood, and their attendance invited.

An annual statement, financial and œconomical, of the Hospital, is distributed among the subscribers—and, during the two last years, registers of all the cases have been kept, in which the several particulars of age, profession, residence, disease, duration of treatment and result, are recorded—from these a statistical report for the year 1836 was drawn up by the author of the present communication and published in the 6th volume of the "Camb. Phil. Transactions;" a similar report has been prepared for the year 1837.

The reports will be confined principally to cases that occurred during the years 1836-37, and will be preceded by a short statement, chiefly statistical, of the comparative prevalence, &c., of each disease in the general practice of the hospital, derived from the registers; but the substance of each report will be limited to the contingent of cases which fell to the share of the author, and constructed entirely from an analysis of the notes taken of them in more or less detail, according to their respective importance, or the opportunities afforded for observation. They include the cases of the out as well as the in-patients.

